# Propagating wellness: understanding the communication mechanisms of yoga and Wuqinxi (five animal frolics) via Lasswell’s 5W framework

**DOI:** 10.3389/fpubh.2026.1850942

**Published:** 2026-07-23

**Authors:** Yong Song, Lei Lu, Hanyun Luo

**Affiliations:** 1 School of Foreign Languages, Anqing Normal University, Anqing, China; 2 School of Physical Education, Anqing Normal University, Anqing, China; 3 School of Artificial Intelligence and Computer Science, Anqing Normal University, Anqing, China

**Keywords:** cross-cultural communication, exercise type, Lasswell’s 5W model, practitioners’ consumption behavior, Wuqinxi (five animal frolics), yoga

## Abstract

Mind–body practices are increasingly used in public health promotion and cross-cultural communication, yet comparative evidence on how different practices are disseminated remains limited. This study used a sequential mixed-methods design to compare yoga, a mature transnational practice, with Wuqinxi (Five Animal Frolics), a traditional Chinese practice that has also gained international recognition but remains more regionally concentrated in its grassroots practice. Semi-structured interviews with yoga studio managers, a Wuqinxi inheritor, and practitioners informed questionnaire development and interpretation; the quantitative phase analyzed 389 valid survey responses from yoga (*n* = 210) and Wuqinxi (*n* = 179) practitioners through regression, mediation, and moderation models guided by Lasswell’s 5W framework. Content diversity showed the largest positive association with dissemination effectiveness (*B* = 0.6464), followed by instructor professionalism (*B* = 0.5222), venue facilities (*B* = 0.3706), and channel modernization (*B* = 0.1793). Practitioners’ consumption behavior served as a partial statistical mediator, with direct pathways accounting for 81% of the instructor effect, 79% of the content effect, 60% of the venue effect, and 51% of the channel effect. Exercise type moderated the associations for instructor professionalism, content diversity, and channel modernization: corresponding coefficients were stronger for Wuqinxi than for yoga, whereas venue-facility moderation was unsupported. The main contribution is a bounded 5W-based comparison showing that Wuqinxi dissemination may benefit most from professionalized instruction, diversified content, and modern communication channels, while consumption behavior serves as a limited statistical mediator of engagement. This contributes to cross-cultural communication research by revealing how a transnational practice and an indigenous practice differ in dissemination mechanisms, offering a transferable framework for comparing health communication across cultural contexts.

## 1 Introduction

Sustainable Development Goal 3.4 (SDG 3.4), established by the United Nations, aims to reduce premature mortality from non-communicable diseases (NCDs) by one-third and promote mental health and wellbeing by 2030 (1). Within this global public health framework, there is growing emphasis on the development of low-cost, scalable, and accessible health interventions. Among these, mind–body practices have attracted increasing scholarly and policy attention due to their demonstrated benefits in chronic disease prevention, psychological stress reduction, and overall improvement of quality of life (2). Compared to conventional pharmaceutical and medical treatments, these practices offer lower entry barriers and greater adaptability, making them a promising avenue for advancing the SDG 3.4 agenda.

Yoga, in particular, has gained global prominence, with an estimated 300 million practitioners worldwide. It is widely integrated into chronic disease management, mental health promotion, and community wellness programs (3–5). In contrast, traditional Chinese mind–body practices—such as Tai Chi, Wuqinxi (Five Animal Frolics), and Baduanjin—though comparable in their theoretical foundations and health benefits, remain underutilized both domestically and globally. This disparity reflects a cultural asymmetry in dissemination capacity and highlights the urgency of incorporating indigenous health resources into public health promotion strategies. As China advances its Healthy China 2030 agenda, understanding and addressing the dissemination gap of traditional practices like Wuqinxi becomes increasingly essential.

This study focuses on Wuqinxi, a representative form of traditional Chinese mind–body exercise with a documented history spanning more than 1,800 years. Originating in Bozhou, Anhui Province during the Eastern Han Dynasty, Wuqinxi was developed by the renowned physician Hua Tuo and embodies not only distinctive cultural characteristics rooted in Traditional Chinese Medicine (TCM) but also the holistic philosophy of “Dao follows nature,” which emphasizes harmony between the human body and the natural world (6). It is the world’s first complete set of animal-imitating qigong exercises (7). Its movements are inspired by five animals, namely the tiger, deer, bear, monkey, and crane (6), and are believed to harmonize the five zang organs (viscera in Traditional Chinese Medicine): the Tiger exercise soothes the liver, the Deer exercise tonifies the kidneys, the Bear exercise regulates the spleen and stomach, the Monkey exercise nourishes the heart, and the rane exercise invigorates the lungs (8). Grounded in both somatic and meditative traditions, Wuqinxi emphasizes the coordinated regulation of movement, breathing, and mental focus, reflecting a holistic orientation similar to yoga (9, 10).

Wuqinxi is not only a traditional Chinese practice but also an internationally studied mind–body intervention with a well-established evidence base. An expanding body of empirical research has demonstrated the physiological and psychological benefits of Wuqinxi. In terms of chronic diseases, Wuqinxi has shown preventive and therapeutic effects on conditions such as Parkinson’s disease (11), arthritis (12), insomnia (13), chronic low back pain (14, 15), osteoporosis (16) and ankylosing spondylitis (17). In terms of mental health, Wuqinxi has shown clear potential in alleviating depression and anxiety (18–20). Furthermore, studies indicate that Wuqinxi may enhance attentional control in children (21). Given that attention deficit is the predominant subtype of learning disabilities among students (22), this finding further highlights the broad relevance of Wuqinxi in promoting population-wide health.

A distinctive advantage of Wuqinxi lies in its minimal resource requirements. It can be practiced without specialized equipment, dedicated venues, or technical attire, and is often taught through in-person demonstration and oral instruction, with public spaces such as parks and community squares serving as the main practice venues. Research confirms that urban green spaces are significantly positively correlated with quality of life (23). The promotion of Wuqinxi can further amplify the health-enabling role of such public resources. These characteristics make it highly adaptable to resource-constrained settings and accessible to vulnerable populations, including older adults and low-income communities (24). Compared with yoga—which often involves institutionalized settings, standardized equipment, and market-oriented instruction—Wuqinxi offers a low-cost, inclusive alternative that aligns well with public health goals such as reducing health disparities and expanding universal access to preventive care.

Despite its long history and documented health benefits, the dissemination of Wuqinxi presents a distinctive pattern: wide administrative promotion at home and abroad coexists with concentrated grassroots practice in a few regions. Nationally, under the coordination of the National Sports Administration’s Health Qigong Management Center, Wuqinxi has been promoted across all 31 provinces, autonomous regions, and municipalities, with local governments supporting teaching and promotional activities (25). Internationally, coordinated by the International Health Qigong Federation (IHQF), Wuqinxi has now spread to over 50 countries and regions, with teaching and exchange activities conducted in countries such as Germany, the United Kingdom, France, the United States, Austria, the Netherlands, Peru, and Argentina (25, 26). Yet despite this broad administrative reach, sustained grassroots practice remains heavily concentrated in Anhui and Jiangsu, the birthplace and surrounding areas, reflecting an uneven dissemination pattern. This imbalance indicates a communication and public health dissemination gap. Existing evidence has established the potential health value of Wuqinxi, yet less is known about how communicator capacity, content design, channel modernization, and engagement-related behavior are associated with dissemination effectiveness. Framing the problem in this way shifts attention from whether Wuqinxi can improve health under practice conditions to how a low-cost indigenous practice can reach and retain practitioners in everyday community settings.

The classic communication model proposed by Lasswell emphasizes the key elements of source, message, channel, audience, and effect (27). McQuail notes that these elements form the basis of the 5W framework (28), which provides a systematic lens for analyzing communication processes (27). The present study adopts Lasswell’s 5W model as the core analytical framework. This model—comprising the components *Who* (communicator), *What* (message content), *Which Channel* (medium), *To Whom* (audience), and *With What Effect* (communication outcomes)—provides a structured lens through which to analyze the dissemination pathways of Wuqinxi and yoga. Prior research has explored individual dimensions of the 5W model within specific contexts. For example, Siegel et al. (29) examined the multi-stakeholder and regionally differentiated architecture of yoga certification systems (*Who*), Coskuner-Balli and Ertimur (30) analyzed the cultural adaptation of yoga to meet localized consumer preferences (*What*), and Li et al. (31) investigated the dual role of digital media in both amplifying yoga’s reach and spreading misinformation (*Which Channel*). In the context of Wuqinxi, Zhang (32) identified governments, educational institutions, and cultural inheritors as key disseminators (*Who*), while Zhang et al. (33) emphasized the importance of combining online community-building with offline engagement (*Which Channel*).

However, current research exhibits significant limitations. First, existing work predominantly consists of fragmented, isolated studies of individual elements, lacking integrative analysis of how different communication factors collectively influence dissemination outcomes. Furthermore, few studies have considered the moderating role of exercise type in shaping the relationship between communication elements and their effects. This gap constrains a holistic understanding of dissemination mechanisms across culturally and structurally distinct practices. Second, there is a pronounced imbalance in the depth and breadth of research between the two practices. Research on Yoga is not only abundant but also more detailed, in-depth, and closely aligned with contemporary developments: its scope extends to factors influencing instructor selection and measurement scales (34), the effectiveness of instructors’ online teaching (35); socio-demographic characteristics of different practitioner groups (36–38), and even barriers to participation among specific groups such as men (39) and pregnant women (40); studies on Yoga dissemination outcomes encompass health, psychological, and economic dimensions (41–43); simultaneously, research maintains a cutting-edge focus on emerging online dissemination models (44–46) and the application of smart IoT technologies (47). In contrast, current research on Wuqinxi focuses predominantly on its therapeutic effects, lacking in-depth and categorized studies on its communicators and practitioners. There is also an absence of market-oriented research on content adaptation. While some studies have begun to explore the digital transformation (48) and AI applications of Wuqinxi (49), their number remains exceedingly limited. This stark contrast reflects both inadequate dissemination and research into Wuqinxi itself.

The 5W framework is used here for its structured categories that facilitate comparison between two practices with markedly different dissemination systems. At the same time, we explicitly acknowledge its limitations, which bound the scope of our claims. The model’s static categories do not capture reciprocal interaction, iterative feedback, or socio-cultural context (50, 51); therefore, the present analysis does not claim to reconstruct a dynamic communication system. It uses the model to organize supply-side factors that are visible to dissemination actors: instructor professionalism, content diversity, channel modernization, and venue facilities. Consumption behavior is treated only as a limited indicator of engagement and resource commitment, and the excluded audience component remains an explicit limitation. This bounded use is consistent with scholarship that treats Lasswell’s model as a flexible heuristic for communication analysis (52). Alternative dissemination frameworks, including diffusion of innovations and RE-AIM, emphasize adoption, implementation, maintenance, and public health impact across broader systems (53, 54). The present study retains the 5W model because its categories align directly with the measured communicator, content, channel, access, and perceived-effect variables.

This study uses yoga as the comparative case for Wuqinxi for three reasons. First, both practices are non-pharmacological mind–body exercises involving coordinated movement, breath regulation, and embodied instruction, which makes dissemination comparison meaningful at the level of practice type. Second, the two practices occupy sharply different dissemination systems. While both have achieved transnational reach, yoga has developed commercial studios, transnational certification, branded content variants, digital instruction, and a comparatively heterogeneous practitioner base, whereas Wuqinxi remains more closely tied to public cultural institutions, local inheritors, community practice sites, and geographically concentrated audiences in Anhui and Jiangsu, and has not been commercialized to the same extent as yoga. Third, Wuqinxi’s combination of documented health value, low resource requirements, and limited diffusion creates a public health rationale for identifying which communication elements appear most strongly associated with dissemination effectiveness. The analysis treats yoga and Wuqinxi as two practices with markedly different dissemination configurations, and the comparison is used to identify bounded, interpretable differences.

This study advances prior research in four respects. First, it frames the comparison as a communication problem within public health dissemination and examines how supply-side communication elements are associated with perceived dissemination effectiveness. Second, it treats practitioners’ consumption behavior as a self-reported indicator of sustained engagement and resource commitment, which links market-related participation to dissemination capacity without equating consumption with health benefit. Third, it evaluates exercise type as a contextual moderator that captures structural differences between a mature transnational practice and a regionally concentrated indigenous practice. Fourth, it contributes to cross-cultural communication research by revealing how a transnational practice and an indigenous practice differ in dissemination mechanisms, offering a transferable framework for comparing health communication strategies across cultural contexts. These contributions are bounded by the cross-sectional design and by non-equivalent sampling contexts, so the analysis supports interpretation of associations and comparative patterns, with causal mechanisms reserved for future longitudinal or experimental work.

## 2 Theoretical model and hypotheses

### 2.1 Theoretical model

This study adopts Lasswell’s 5W model (27) as its core analytical framework. The model conceptualizes communication through five interrelated components: Who (communicator), Says What (message content), Through Which Channel (medium or access route), To Whom (audience), and With What Effect (outcome). It has been widely applied in interdisciplinary research domains (55). In public health, for instance, Landry and Kacou (56) used the model to inform epidemic prevention strategies; Lin et al. (57) and Wei et al. (50) applied it to trace the dynamics of online public opinion and the dissemination of health-related misinformation during epidemics. Dong et al. (58) further explored how 5W components moderate the effectiveness of rumor correction efforts. Wang and Cai used this model to study the implementation of health education in urban communities in China (59). Zeng used the model to investigate mass health communication through the WeChat official account “Dingxiang Doctor.” (60) In the domain of exercise communication, the model has been employed to analyze sports-related social media discourse (61) and to examine the cross-cultural dissemination of traditional Chinese practices such as martial arts (62) and Tai Chi (63), demonstrating its adaptability across disciplinary and contextual boundaries. In the present study, its function is classificatory and analytic: it organizes observable supply-side factors and guides tests of their associations with perceived dissemination effectiveness.

Operationally, instructor professionalism represents Who, content diversity represents Says What, channel modernization represents mediated channels, and venue facilities represent the physical access conditions through which embodied instruction is delivered. Dissemination effectiveness represents With What Effect. Practitioners’ consumption behavior is included as a self-reported engagement and resource-commitment indicator, which allows the analysis to test a statistical indirect pathway between communication factors and perceived dissemination effectiveness. This variable does not constitute a real-time feedback loop or a full measure of audience response.

The To Whom component is acknowledged as a boundary of the study. Audience characteristics were not modeled as a separate empirical dimension because the research prioritizes modifiable supply-side factors for dissemination strategy, and because available data do not support precise segmentation of needs, barriers, or differential responses. Consequently, the findings are most informative for supply-side communication design and should not be interpreted as a complete account of audience heterogeneity. Diffusion and RE-AIM perspectives remain useful comparators for future work because they attend to adoption, reach, implementation, and maintenance at audience and institutional levels (53, 54). This scope also limits the theoretical contribution to a bounded application of the 5W framework in comparative mind–body practice dissemination.

### 2.2 Hypothesis formulation

#### 2.2.1 Communicator hypothesis

Communicators are central to the dissemination of embodied health practices because instructor competence shapes instructional clarity, perceived safety, and practitioner experience (64). Professional guidance may strengthen identification with a practice and support sustained engagement over time (65). In cross-sectional terms, this study therefore expects instructor professionalism to be positively associated with perceived dissemination effectiveness, because practitioners who report higher instructional quality are more likely to report satisfaction, trust, and willingness to recommend or continue the practice.

Yoga’s global proliferation has been supported by mature, multi-tiered certification systems administered by organizations such as Yoga Alliance, the International Association of Yoga Therapists (IAYT), and the Yoga Certification Board (YCB), with more than 100,000 certified instructors across over 100 countries (29). In contrast, Wuqinxi dissemination remains largely state-led through institutions such as the China Health Promotion and Education Association (CHQA), with internationally certified instructors still relatively limited, estimated at 3,000–5,000 (66).

Practitioners’ consumption behavior is included as a statistical mediator, and the analysis does not treat it as evidence of a proven causal mechanism. The theoretical expectation is that favorable communication elements may be accompanied by stronger value recognition and willingness to maintain participation, which may appear in purchase-related behavior such as attire, equipment, or instructional materials (see Q16–Q18 in Table 1) (67). In this design, such behavior is interpreted as a self-reported indicator of engagement and resource commitment. The mediation tests therefore estimate whether an indirect association through consumption behavior is present alongside the direct association between each communication element and perceived dissemination effectiveness.

**Table 1:** Survey questionnaire on factors and mechanisms influencing the dissemination of Wuqinxi and yoga.

Variables and Indicators		Measurement Items
Control variables	Personal characteristics	Your gender
		Your age
		Your occupation
Independent variables	Professionalism of instructor	Q1 Have the instructor at your gym received professional training?
		Q2 Does the instructor at your gym hold instructor certification?
		Q3 Does the instructor at your gym have over 5 years of teaching experience?
		Q4 Are the instructor at your gym full-time professionals?
		Q5 Does the instructor at your gym undergo regular training and evaluations?
	Adequacy of venue facilities	Q6 Does your gym provide comprehensive basic facilities, including changing rooms, showers, restrooms, etc.?
		Q7 Does your gym feature comprehensive auxiliary facilities, including rest areas, drinking fountains, storage spaces, and emergency equipment?
		Q8 How would you rate the maintenance of your gym’s facilities (e.g., cleanliness, equipment availability)?
	Diversity of content offered	Q9 Do you feel your gym offers a wide variety of classes?
		Q10 Does your club’s teaching content include technical movements?
		Q11 Does your club’s technical movement instruction feature diverse combinations tailored to specific teaching objectives, rather than fixed routines?
		Q12 Does your club’s teaching content cover the theoretical principles, health benefits, and characteristics of each movement?
		Q13 Does your center’s curriculum include breathing techniques and mental focus?
		Q14 Does your center’s curriculum include content beyond the above (technical movements, principles/benefits/characteristics of each form, breathing, mental focus)?
	Modernization of communication channels	Q15 Did you learn about this program at your center through modern media like the internet or social media?
Mediator variables	Practitioner’s consumption behavior	Q16 Have you purchased basic products related to this program (e.g., practice attire)?
		Q17 Have you purchased advanced products related to this program (e.g., training equipment)?
		Q18 Have you purchased instructional products related to this program (e.g., books, video materials)?
Dependent variables	Effectiveness of exercise dissemination	Q19 Are you satisfied with the courses offered by your gym?
		Q20 Practicing this program has significantly improved your physical fitness
		Q21 Practicing this program enriches your leisure time
		Q22 Practicing this program has expanded your social circle
		Q23 Practicing this program fulfills your pursuit of beauty and fashion
		Q24 Practicing this program effectively relieves your stress and improves your mood

Exercise type is used as a contextual moderator because yoga and Wuqinxi differ in certification maturity, institutional support, commercial infrastructure, and audience reach. The moderation hypotheses therefore test whether the strength of the associations varies between Wuqinxi and yoga; they do not establish that exercise type itself causes those differences. Accordingly, the following hypotheses are proposed:

*H1a (main association)*: Instructor professionalism is positively associated with perceived dissemination effectiveness.

*H1b (statistical mediation)*: Practitioners' consumption behavior forms a statistical indirect pathway between instructor professionalism and perceived dissemination effectiveness.

*H1c (moderation)*: Exercise type moderates the association between instructor professionalism and perceived dissemination effectiveness, with a stronger association expected for Wuqinxi than for yoga.

#### 2.2.2 Venue facilities hypothesis

Venue facilities provide physical access conditions for both exercise-related information and embodied practice. Adequate facilities may improve accessibility, comfort, and safety, thereby contributing to perceived service value. These characteristics can be associated with willingness to invest time and resources in continued practice and with peer recommendation (68). From a public health perspective, the physical environment of practice sites shapes the feasibility of repeated participation and the potential scope of population coverage.

In China, Wuqinxi is primarily promoted through public welfare channels, such as fitness qigong sites supported by local governments and institutions like the sports bureau. Approximately 2,200 new Wuqinxi practice sites have been established over the past decade (69), most of which are located in public outdoor spaces such as parks and public squares. While such locations enhance physical accessibility, they often lack basic amenities, including restrooms, showers, and storage areas. In contrast, yoga is disseminated predominantly through market-based institutions such as yoga studios and fitness clubs, which typically operate on a fee-for-service model. This commercial orientation allows for investment in well-equipped, climate-controlled indoor venues with standardized service infrastructure (e.g., locker rooms, waiting areas), thereby contributing to a more structured and immersive experience and higher participant retention.

Therefore, the following hypotheses test associations and statistical indirect pathways:

*H2a (main association)*: The adequacy of venue facilities is positively associated with perceived dissemination effectiveness.

*H2b (statistical mediation)*: Practitioners' consumption behavior forms a statistical indirect pathway between venue-facility adequacy and perceived dissemination effectiveness.

*H2c (moderation)*: Exercise type moderates the association between venue facilities and perceived dissemination effectiveness, with a stronger association expected for yoga than for Wuqinxi.

#### 2.2.3 Communication content hypothesis

Communication content represents the Says What component and is expected to shape dissemination through cognitive, motivational, and behavioral engagement: information can increase understanding, motivate practice, and support continued participation (70). Diverse and professional content, including technical instruction, health education, and lifestyle guidance, may increase informative value, emotional relevance, and self-efficacy. The study therefore treats content diversity as a communication factor expected to be positively associated with perceived dissemination effectiveness.

Yoga has evolved a wide range of content variations to meet diverse practitioner needs, such as Bikram Yoga’s emphasis on physical intensity and Jivamukti Yoga’s focus on ethical and spiritual dimensions (30). Its instruction often integrates mindfulness (71), relaxation techniques (72), and dietary principles (73). In contrast, Wuqinxi instruction typically prioritizes the demonstration of physical forms and places less emphasis on theoretical frameworks or holistic health narratives (32). Based on this contrast, content diversity is expected to show a positive association with perceived dissemination effectiveness overall, with a stronger association for Wuqinxi, where baseline content diversity is relatively limited, than for yoga, where additional diversification may yield smaller marginal gains.

Based on this rationale, this study proposes the following hypotheses:

*H3a (main association)*: Content diversity is positively associated with perceived dissemination effectiveness.

*H3b (statistical mediation)*: Practitioners' consumption behavior forms a statistical indirect pathway between content diversity and perceived dissemination effectiveness.

*H3c (moderation)*: Exercise type moderates the association between content diversity and perceived dissemination effectiveness, with a stronger association expected for Wuqinxi than for yoga.

#### 2.2.4 Communication channels hypothesis

Communication channels represent the means through which practice information reaches and engages potential participants. Modern platforms, particularly digital and social media, offer audience targeting, low marginal distribution costs, and network spillover; these features may be associated with wider reach, recurring interaction, and sustained participation. This study therefore expects channel modernization to be positively associated with perceived dissemination effectiveness.

Yoga has already capitalized extensively on online and social media ecosystems, thus achieving cross-regional diffusion and rapid growth during the pandemic due to its convenience, affordability, and psychological benefits (31, 74–76). By contrast, Wuqinxi continues to rely largely on offline channels such as community programs, school-based initiatives, and traditional broadcast media, which are often led by government agencies (24). Integrating Wuqinxi into digital platforms such as Douyin and RedNote (Xiaohongshu) may enhance its interactivity and novelty, thus extending its reach to new audiences and stimulating greater participation. Given yoga’s already mature online–offline model, the marginal gains from further digital investment are hypothesized to be more limited, whereas Wuqinxi holds greater potential for dissemination improvement through digital channel modernization.

Based on the above analysis, this study proposes the following hypotheses:

*H4a (main association)*: Communication channel modernization is positively associated with perceived dissemination effectiveness.

*H4b (statistical mediation)*: Practitioners' consumption behavior forms a statistical indirect pathway between communication channel modernization and perceived dissemination effectiveness.

*H4c (moderation)*: Exercise type moderates the association between communication channel modernization and perceived dissemination effectiveness, with a stronger association expected for Wuqinxi than for yoga.

**Figure 1:**
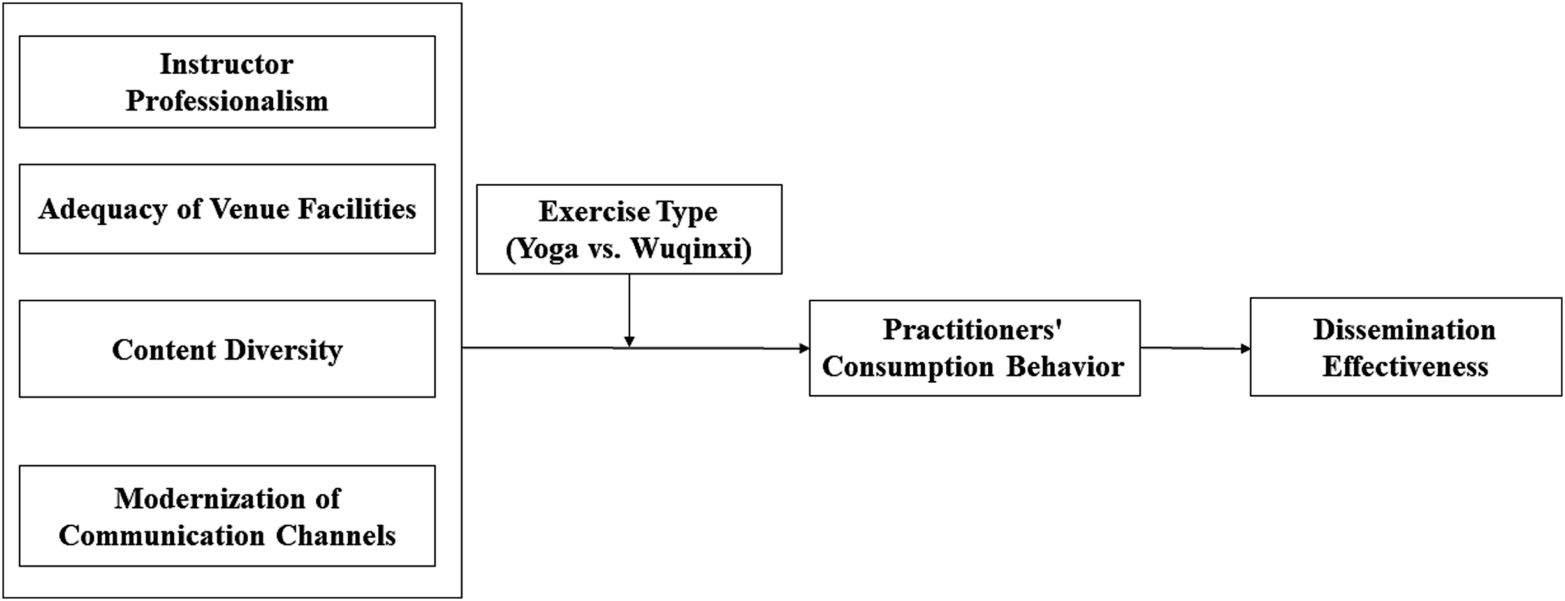
Research model. This model maps instructor professionalism to who, content diversity to says what, channel modernization to mediated channels, and venue facilities to physical access conditions for practice-based communication. Dissemination effectiveness represents with what effect. Practitioners’ consumption behavior is modeled as a limited statistical mediator indicating engagement and resource commitment, and exercise type (yoga or Wuqinxi) is modeled as a moderator of selected associations. The To Whom component is acknowledged as outside the empirical model.

The research model of this study is shown in Figure 1.

## 3 Methods and data

Participants were involved through anonymous questionnaires and semi-structured interviews in a low-risk study. Before completing the questionnaire, respondents were informed of the study purpose, the voluntary nature of participation, and the anonymous handling of responses, and consent was obtained. Interviewees were informed of the research purpose, consented to the academic use of their responses, and were anonymized in reporting. No sensitive personal data were collected, confidentiality was maintained during data handling, and no experimental intervention was administered. According to institutional regulations, formal ethical approval was waived.

### 3.1 Data collection

#### 3.1.1 Questionnaire design

This study explores the factors and mechanisms associated with the dissemination of Wuqinxi, a traditional Chinese fitness practice, by comparing its communication strategies with those of yoga. A sequential mixed-methods design was adopted in which the qualitative phase preceded the survey phase. The qualitative component was used to refine the empirical constructs, adapt questionnaire items to the two practice contexts, and provide contextual interpretation of the quantitative results; the quantitative component then tested associations among communication factors, practitioners’ consumption behavior, and perceived dissemination effectiveness.

The qualitative sample was purposive. Informants were selected because they represented complementary positions in the dissemination process: institutional yoga dissemination, Wuqinxi inheritance, and practitioner experience in cities where Wuqinxi teaching and community practice were accessible. The researchers first visited three chain yoga studios, Private Yoga, Bo Fan Yoga, and Seek Yoga Tree, and interviewed management staff about yoga studio organization, instructor credentials, course content, and dissemination channels in China. They then interviewed a 58th-generation inheritor of Wuqinxi, referred to as Interviewee ZH for anonymity, and three practitioners from Nanjing, Anqing, and Bozhou. Interview topics were aligned with the 5W framework and focused on communicator credibility, content forms, channel use, venue conditions, and practitioner engagement. The analytic treatment was pragmatic and framework-guided: interview information and field observations were reviewed against the 5W categories to identify recurring practical concerns for questionnaire development and later interpretation. Integrating these insights with scholarly references from Mandlik et al. (77), van de Oudeweetering et al. (78), and Li (79), the researchers developed a questionnaire based on Lasswell’s communication model (Table 1). From an initial pool of 50 items, 27 were retained for the final questionnaire to ensure alignment with the core constructs under investigation. Of these 27 items, 3 were demographic multiple-choice items used to collect basic information, and 24 were five-point Likert-scale items (1 = Strongly Disagree to 5 = Strongly Agree) designed to measure the study constructs. Items were scored such that higher scores indicated stronger agreement. For data analysis, a composite score for each construct was computed by averaging its constituent items. The interviews supported instrument development and contextual explanation. Accordingly, the manuscript limits qualitative claims to design rationale and cautious interpretation in the absence of a separately coded qualitative results section.

The construct labels are interpreted narrowly because all substantive variables were measured with five-point self-report items. Dissemination effectiveness denotes perceived satisfaction and participation effects rather than objective diffusion, clinical effectiveness, or verified reach. Practitioners’ consumption behavior denotes reported purchases of practice products, equipment, and instructional materials, so it is a purchase-related engagement indicator rather than a comprehensive measure of market size, participation frequency, or health behavior. The regression estimates are therefore interpreted as associations among perceived constructs.

#### 3.1.2 Sampling strategy and data collection

The questionnaire was distributed through online and offline channels. According to industry data from Miliyue (2024), Chinese yoga studios are distributed as follows: 53% in Eastern China, 25% in Central China, and 19% in Western China. Therefore, this study commissioned the QuestionStar survey platform to distribute questionnaires to practitioners in yoga studios in Guangdong and Jiangsu in Eastern China, Anhui in Central China, and Shaanxi in Western China. Of 276 questionnaires, 210 were valid: 59 from Guangdong, 54 from Jiangsu, 48 from Anhui, and 49 from Shaanxi. This plan provided regional spread within the available yoga sampling frame; however, it remains a targeted practitioner sample rather than a national probability sample.

Given the concentrated dissemination of Wuqinxi in Anhui and Jiangsu, this study first used Tencent’s survey platform to distribute questionnaires in Anhui, Jiangsu, Beijing, and Shanghai; 75 of 88 responses were valid, including 50 from Anhui, 12 from Jiangsu, 7 from Beijing, and 6 from Shanghai. In addition, 50 questionnaires were distributed on-site at the Wuqinxi teaching venue in the Nanjing Workers’ Cultural Palace (Jiangsu Province), yielding 37 valid responses, and 80 questionnaires distributed through Interviewee ZH yielded 67 valid responses. In total, 179 of 218 Wuqinxi questionnaires were valid. This procedure weighted the sample toward core Wuqinxi dissemination sites rather than a geographically balanced national sample. Table 2 shows the basic characteristics of the surveyed subjects.

**Table 2:** Descriptive statistics of the sample.

Basic Information	Category	Number (persons)	Percentage (%)
Gender	Male	140	35.99
	Female	249	64.01
Age	15 years and below	0	0.00
	16–25 years	53	13.62
	26–35 years old	166	42.67
	36–45 years old	78	20.05
	46–55 years old	38	9.77
	56–65 years old	44	11.31
	66 years old and above	10	2.57
Occupation	Government employees	27	6.94
	Corporate employees	214	55.01
	Medical professionals	13	3.34
	Educators	19	4.88
	Business professionals	17	4.37
	Farmers	6	1.54
	Students	22	5.66
	Retirees	38	9.77
	Other	33	8.48

The geographic concentration of Wuqinxi respondents in Anhui and Jiangsu is therefore a design constraint and an interpretation limit. It supports analysis in active dissemination contexts where teaching resources, inheritor networks, and community practice venues are accessible. In contrast, estimates should not be generalized directly to regions where Wuqinxi is less institutionalized or less visible. Yoga-Wuqinxi comparisons should therefore be read as comparisons between structurally different dissemination settings generated from non-equivalent sampling frames.

The sample also varied by age and occupation, yet descriptive tests showed age and gender differences between yoga and Wuqinxi practitioners (see Section 3.2 for detailed statistics). Consequently, robustness checks included age, gender, and occupation as covariates to evaluate stability after adjustment for measured demographic heterogeneity. These controls reduce one source of bias, while unmeasured regional and institutional differences remain relevant to interpretation.

### 3.2 Data pre-tests

#### 3.2.1 Common method bias

To mitigate potential common method bias, respondents were assured of anonymity to reduce evaluation apprehension and socially desirable response bias. Harman’s single-factor test showed that the first unrotated factor explained 38.33% of the total variance, below the conventional 50% threshold (80). This result does not indicate a dominant single factor, although it remains a diagnostic check rather than proof that all common-method risk has been eliminated.

#### 3.2.2 Reliability and validity

As shown in Table 3, the diagnostics supported use of the questionnaire composites while remaining limited. Cronbach’s alpha was 0.883, indicating strong internal consistency. The KMO value was 0.945, and Bartlett’s test of sphericity was significant (*p* < 0.001), supporting factorability of the item matrix. These diagnostics support internal coherence and factor-analysis suitability, but they do not establish objective construct validity.

**Table 3:** Results of reliability and validity tests.

Test category	Measurement metric	Statistical value
Reliability	Number of questionnaire items	27
	Cronbach’s *α*	0.883
Validity	KMO measure of sampling adequacy	0.945
	Bartlett’s test of sphericity	*χ*^2^ = 4335.609, df = 351, ^**^p < 0.001

#### 3.2.3 Multicollinearity diagnostics

Multicollinearity was evaluated using variance inflation factors (VIF). In the main-effect models, the VIF values were 1.80 for instructor professionalism, 1.31 for modernization of communication channels, 1.82 for content diversity, and 1.70 for adequacy of venue facilities. All values are well below the commonly used threshold of 5.0, suggesting that multicollinearity is not a serious issue and is unlikely to distort the regression estimates.

#### 3.2.4 Between-group demographic comparison

Descriptive tests showed age differences (*t*(387) = 6.68, *p* < 0.001) and gender-composition differences (*χ*^2^(1) = 19.96, *p* < 0.001) between yoga and Wuqinxi practitioners. Age, gender, and occupation were included as robustness-check covariates.

### 3.3 Research process

This study analyzed 389 valid questionnaire responses using OLS regression, statistical mediation tests, and moderation models. The dependent variable was self-reported perceived dissemination effectiveness, and four independent variables were examined: instructor professionalism, adequacy of venue facilities, content diversity, and channel modernization. Self-reported purchase-related consumption behavior was specified as a statistical mediator to evaluate indirect associations, while exercise type (Wuqinxi = 0, Yoga = 1) was specified as a contextual moderator to examine whether associations varied across practice forms.

The empirical analysis proceeded in three stages. First, ordinary least squares regression was applied to estimate associations between each independent variable and perceived dissemination effectiveness. Second, mediation was examined through a stepwise regression approach that estimated the association between each independent variable and the dependent variable, the association between each independent variable and the mediator, and the joint associations of the independent variable and mediator with the dependent variable. These models identify whether coefficients are consistent with indirect associations; because the data are cross-sectional, they do not prove temporal ordering or causal mediation. Third, moderation was tested by introducing interaction terms between each independent variable and exercise type to examine whether the strength or direction of associations differed across practice types.

## 4 Results

### 4.1 Hypothesis testing for instructor

#### 4.1.1 Overall effect

Table 4 shows a positive association between instructor professionalism and perceived dissemination effectiveness (*B* = 0.5222, 95% CI: 0.472–0.572). This coefficient is the second largest among the four main predictors, below content diversity and above venue facilities and channel modernization. The estimate supports H1a and indicates that instructor quality is a comparatively strong correlate of perceived dissemination effectiveness.

**Table 4:** Summary of overall effects of four independent variables on dissemination effectiveness.

IV	*B*	SE	*t*-value	*p*	95%	CI
Instructor professionalism	0.5222^***^	0.025	20.583	0.000	0.472	0.572
Venue facilities	0.3706^***^	0.027	13.903	0.000	0.318	0.423
Content diversity	0.6464^***^	0.033	19.655	0.000	0.582	0.711
Channels modernization	0.1793^***^	0.021	8.590	0.000	0.138	0.220

IV, Independent Variable; B, Non-standardized Coefficient; SE, Standard Error; p, Significance; 95% CI, 95% Confidence Interval.

The results in the table are derived from four separate simple linear regression analyses. The constants for each model are as follows: 2.0907 for the Instructor Professionalism model, 2.7195 for the Venue Facilities model, 1.5708 for the Content Diversity model, and 3.5733 for the Channels Modernization model. ^***^ indicates *p* < 0.001.

See Supplementary Tables S1–S4 for full results.

#### 4.1.2 Mediating effect

Table 5 shows that instructor professionalism was positively associated with practitioners’ consumption behavior (*a* = 0.6881) and that consumption behavior was positively associated with dissemination effectiveness after instructor professionalism was included in the model (*b* = 0.1429). The direct coefficient decreased from 0.5222 to 0.4238 after the mediator was added. The implied indirect association was approximately 0.0984, or about 19% of the total association.

**Table 5:** Summary of the mediating effects of practitioners’ consumption behavior between four independent variables and dissemination effectiveness.

IV	c (total effect)	a (IV → M)	b (M → Y)	c′ (direct effect)
Instructor professionalism	0.5222^***^	0.6881^***^	0.1429^***^	0.4238^***^
Venue facilities	0.3706^***^	0.5747^***^	0.2596^***^	0.2215^***^
Content diversity	0.6464^***^	0.8547^***^	0.1612^***^	0.5086^***^
Channels modernization	0.1793^***^	0.2522^***^	0.3501^***^	0.0910^***^

IV, Independent Variable; M, Consumption (mediator variable); Y, Dissemination Effectiveness. For each independent variable: c, total effect of IV on Y; a, effect of IV on M; b, effect of M on Y while controlling for IV; c′, direct effect of IV on Y while controlling for M. All coefficients are unstandardized (B). ^***^p < 0.001.

See Supplementary Tables S5–S8 for full results.

These estimates support H1b as partial statistical mediation. They also show that the direct association remained dominant: after accounting for consumption behavior, the direct effect represented approximately 81% of the total effect (0.4238/0.5222). Consequently, the mediation result should be interpreted as a secondary indirect pathway rather than as the main explanation for the instructor professionalism association.

#### 4.1.3 Moderating effect

Exercise type was coded as Wuqinxi (0) and Yoga (1), so a negative interaction term indicates a weaker association in the Yoga group than in the Wuqinxi group. As shown in Table 6, for instructor professionalism, the interaction coefficient was −0.2315. The adjusted association was 0.5160 in the Wuqinxi group and 0.2845 in the Yoga group, meaning that the Wuqinxi coefficient was approximately 1.8 times the Yoga coefficient. This comparative magnitude supports H1c and indicates that instructor professionalism was more strongly associated with perceived dissemination effectiveness among Wuqinxi practitioners.

**Table 6:** Summary of moderation effects by exercise type on dissemination effectiveness.

IV	Model 1: TE moderation		Model 3: DE moderation		
	TE (c)	IV × W	DE (c′)	IV × W	M → Y (b)
Instructor professionalism	0.5699^***^	−0.1605^*^	0.5160^***^	−0.2315^**^	0.0824
Venue facilities	0.3928^***^	−0.0596	0.2405^***^	−0.0366	0.2944^***^
Content diversity	0.6831^***^	−0.2010^*^	0.6079^***^	−0.2903^**^	0.0934^*^
Channels modernization	0.3223^***^	−0.2737^***^	0.1875^***^	−0.1561^***^	0.3167^***^

IV, Independent Variable; TE, Total Effect (c path); DE, Direct Effect (c′ path); M, Practitioners’ consumption behavior (mediator); Y, Dissemination Effectiveness; W, Exercise Type (Wuqinxi, 0, Yoga, 1); IV × W, interaction term between independent variable and exercise type. c, total effect of IV on Y; c′, direct effect of IV on Y while controlling for M; b, effect of M on Y while controlling for IV; M → Y (b), effect of mediator on Y (b path) while controlling for IV. Model 1 tests whether exercise type moderates the total effect of IV on Y, without controlling for the mediator. Model 3 tests whether exercise type moderates the direct effect of IV on Y (c′ path), while controlling for the mediator. All coefficients are unstandardized (B). ^*^p < 0.05, ^**^p < 0.01, ^***^p < 0.001.

See Supplementary Tables S9–S12 for full results.

### 4.2 Testing facilities -related hypotheses

#### 4.2.1 Overall effect

Table 4 shows a positive association between adequacy of venue facilities and perceived dissemination effectiveness (*B* = 0.3706, 95% CI: 0.318–0.423). The coefficient was moderate relative to the other predictors: smaller than the coefficients for content diversity and instructor professionalism, yet larger than the coefficient for channel modernization. This pattern supports H2a while indicating that venue facilities were not the strongest correlate of perceived dissemination effectiveness in the model.

#### 4.2.2 Mediating effect

Table 5 shows that venue facilities were positively associated with consumption behavior (*a* = 0.5747), and consumption behavior was associated with dissemination effectiveness after venue facilities were included (*b* = 0.2596). The direct coefficient decreased from 0.3706 to 0.2215, leaving an implied indirect association of approximately 0.1491. The direct effect accounted for about 60% of the total association, while the indirect pathway accounted for about 40%. Thus, H2b was supported as partial statistical mediation, with both direct and indirect components contributing to the result.

#### 4.2.3 Moderating effect

Table 6 does not support exercise type moderation for the path from adequacy of venue facilities to dissemination effectiveness (interaction *B* = −0.0366, *p* > 0.05). The adjusted coefficient was 0.2405 for Wuqinxi and 0.2039 (0.2405–0.0366) for Yoga, a small difference compared with the moderated differences observed for instructor professionalism, content diversity, and channel modernization. H2c was therefore rejected. This result suggests that adequate facilities functioned as a shared baseline condition across the two practices rather than as a major source of comparative differentiation.

### 4.3 Hypothesis testing for dissemination content

#### 4.3.1 Overall effect

Table 4 shows that content diversity had the largest positive association with perceived dissemination effectiveness among the four predictors (*B* = 0.6464, 95% CI: 0.582–0.711). Its coefficient exceeded instructor professionalism by 0.1242, venue facilities by 0.2758, and channel modernization by 0.4671. This effect-size ordering supports H3a and identifies content diversity as the strongest correlate of dissemination effectiveness in the main-effect models.

#### 4.3.2 Mediating effect

Table 5 shows that content diversity was strongly associated with consumption behavior (*a* = 0.8547), while consumption behavior remained positively associated with dissemination effectiveness after content diversity was included (*b* = 0.1612). The direct coefficient decreased from 0.6464 to 0.5086, implying an indirect association of approximately 0.1378, or about 21% of the total association. H3b was supported as partial statistical mediation, although the direct association remained the primary component at approximately 79% of the total effect.

#### 4.3.3 Moderating effect

Table 6 shows exercise type moderation for the path from content diversity to perceived dissemination effectiveness (interaction *B* = −0.2903). The adjusted coefficient was 0.6079 in the Wuqinxi group and 0.3176 in the Yoga group, so the Wuqinxi coefficient was approximately 1.9 times the Yoga coefficient. This result supports H3c and indicates that content diversity was more strongly associated with perceived dissemination effectiveness among Wuqinxi practitioners.

### 4.4 Testing communication channels hypotheses

#### 4.4.1 Overall effect

Table 4 shows a positive association between channel modernization and perceived dissemination effectiveness (*B* = 0.1793, 95% CI: 0.138–0.220). This was the smallest main-effect coefficient among the four predictors, indicating a more modest direct association than those observed for content diversity, instructor professionalism, and venue facilities. The estimate supports H4a while showing that channel modernization should be interpreted as a weaker main-effect correlate.

#### 4.4.2 Mediating effect

Table 5 shows that channel modernization was positively associated with consumption behavior (*a* = 0.2522), and consumption behavior had a comparatively large association with dissemination effectiveness after channel modernization was included (*b* = 0.3501). The direct coefficient decreased from 0.1793 to 0.0910, leaving an implied indirect association of approximately 0.0883. The direct and indirect components were therefore similar in size, with the direct effect accounting for about 51% and the indirect pathway about 49% of the total association. H4b was supported as partial statistical mediation, and this was the only predictor for which the indirect component approached the direct component in magnitude.

#### 4.4.3 Moderating effect

Table 6 shows exercise type moderation for the path from channel modernization to dissemination effectiveness (interaction *B* = −0.1561). The adjusted coefficient was 0.1875 in the Wuqinxi group and 0.0314 in the Yoga group. This indicates that channel modernization had a clearer association in the Wuqinxi subgroup. This pattern supports H4c.

### 4.5 Findings

#### 4.5.1 Overall effect

The main-effect coefficients were ordered as follows: content diversity (*B* = 0.6464), instructor professionalism (*B* = 0.5222), venue facilities (*B* = 0.3706), and channel modernization (*B* = 0.1793). The confidence intervals for all four estimates were positive, supporting H1a, H2a, H3 a, and H4a. The coefficient pattern also indicates that content diversity and instructor professionalism were the largest correlates of perceived dissemination effectiveness, whereas channel modernization had a smaller direct association across the four models.

#### 4.5.2 Mediating effect

Practitioners’ consumption behavior partially mediated all four associations, supporting H1b, H2b, H3b, and H4b. The direct effects accounted for approximately 81% of the total effect for instructor professionalism, 79% for content diversity, 60% for venue facilities, and 51% for channel modernization. The indirect proportions were therefore largest for channel modernization and venue facilities, while consumption behavior played a smaller secondary role for instructor professionalism and content diversity.

#### 4.5.3 Moderating effect

Exercise type moderated the associations for instructor professionalism, content diversity, and channel modernization, with stronger coefficients in the Wuqinxi group. The Wuqinxi coefficients were approximately 1.8 times the Yoga coefficient for instructor professionalism, 1.9 times for content diversity, and 6.0 times for channel modernization, noting the very small coefficient in the Yoga group. In contrast, venue-facility moderation was unsupported, with only a 0.0366 coefficient difference between groups. Therefore, the comparative differences were concentrated in professional, content, and channel dimensions rather than in facility adequacy.

#### 4.5.4 5W integration

Within Lasswell’s framework, content diversity represents Says What and instructor professionalism represents Who; these two factors showed the largest associations with perceived dissemination effectiveness. Channel modernization corresponds to Which Channel, with a smaller direct association but a notable indirect component through consumption behavior. Venue facilities functioned as a physical access condition, as exercise type did not moderate this association. Interview material used during instrument development supports this bounded interpretation by identifying practical concerns related to instructor credibility, content interpretation, online-offline channel use, venue access, and practitioner engagement. The qualitative material refined constructs and contextualized findings; it was not analyzed as a separately coded thematic dataset.

### 4.6 Robustness checks

To evaluate the stability of the results, this study conducted robustness checks by adding Age, Gender, and Occupation as control variables to the regression models. This specification also accounts for the demographic differences between yoga and Wuqinxi practitioners identified in Section 3.2. As shown in Supplementary Tables S13–S16, the estimated main effects of the four independent variables remain statistically significant and retain the same directional patterns as in the baseline models, suggesting that the baseline associations were not fully explained by measured demographic heterogeneity.

## 5 Discussion

This study uses the 5W framework to organize observable supply-side conditions and treats consumption behavior as a limited indicator of engagement and resource commitment, comparing self-reported dissemination patterns of yoga and Wuqinxi. The empirical model covers the Who, Says What, Channel, and Effect dimensions, while the audience dimension and dynamic feedback remain outside direct measurement. This clarifies where the 5W model is informative and where it remains incomplete. Hence, Lasswell’s 5W model is used here as a bounded communication heuristic.

The strongest associations were observed for content diversity (*Says What*) and instructor professionalism (*Who*). Channel modernization (*Which Channel*) showed a smaller direct association but a relatively large indirect component through consumption behavior. Venue facilities were positively associated with perceived dissemination effectiveness, although exercise type did not moderate this association. Because the design is cross-sectional and relies on self-reported measures, these results identify communication-related associations and indirect pathways rather than causal mechanisms.

The mobility lens helps explain the core findings of this study. Digital mobility refers to the capacity of instructional and promotional content to move across short-video platforms, social media, and online offline referral channels. This embodies a lightweight, diffusive communication characteristic. Physical mobility refers to the capacity of participants, instructors, and practice routines to move through accessible community sites and training systems. This embodies a presence-based, embodied characteristic. Yoga, as a mature transnational practice, possesses both high digital mobility and high physical mobility. Its content can be easily disseminated through online platforms, and participants can find standardized venues and certified instructors worldwide. In contrast, Wuqinxi dissemination still relies heavily on physical mobility, that is, community sites and in-person instruction in specific regions, while its digital mobility is relatively limited. This difference explains the coefficient pattern observed in this study. Because physical mobility is the primary mode of Wuqinxi dissemination, credible instructors and adaptable content are especially critical for its perceived dissemination effectiveness. Accordingly, the associations for these two factors are stronger for Wuqinxi than for yoga, approximately 1.8 times and 1.9 times the yoga coefficients, respectively. At the same time, the association between channel modernization and dissemination effectiveness is about 6 times larger for Wuqinxi than for yoga, because in a low-digital-mobility environment any digital upgrade, such as using short-video platforms or social media, yields substantial marginal returns.

The interview materials used during instrument development on the one hand identified practical concerns related to instructor credibility, content interpretation, online offline channel use, venue access, and practitioner engagement. On the other hand, they provided contextual depth to the quantitative findings. Semi-structured interviews revealed that yoga studios attract practitioners through formal certification and brand reputation, whereas Wuqinxi practitioners’ trust centers on the inheritor’s personal authority. This helps to explain why instructor professionalism had a stronger association for Wuqinxi. On content, yoga’s diverse offerings already cater to different tastes, while Wuqinxi practitioners tended to desire more explanatory content about health benefits, which supports the finding that content diversity had a stronger association for Wuqinxi. Regarding channels, the interviews found that yoga relies heavily on social media, whereas Wuqinxi depends on community networks. This exactly explains why the association between channel modernization and perceived dissemination effectiveness is much larger for Wuqinxi than for yoga. These qualitative insights do not serve as independent evidence, nor were they analyzed as a separately coded thematic dataset, but they refine constructs and provide contextualization for the findings.

The theoretical contribution is incremental and application-oriented. Thompson and Schulz pointed out that effective health communication is not only based on theoretical frameworks but also focuses on practical application (81). Practical recommendations should follow the observed effect pattern. For Wuqinxi, the largest and strongest comparative associations point first to content diversification and instructor professionalization: content can be diversified through age-appropriate, culturally interpretable modules that integrate health education and breathing techniques; instructor-training programs can strengthen certification, teaching consistency, and explanation of health-oriented practice principles. Channel modernization should support these priorities through short-video, social-media, and online–offline referral content that directs participants to suitable content, credible instructors, and accessible venues. Venue facilities should be maintained as a shared baseline for access, comfort, and repeated participation, rather than framed as the main differentiating policy lever. This interpretation can be read alongside diffusion of innovations and RE-AIM perspectives, because the recommendations distinguish adoption-oriented content, institutional delivery capacity, and maintenance conditions, without reclassifying the present study as an implementation evaluation (53, 54).

Limitations include geographically concentrated sampling of Wuqinxi, reliance on self-reported data, omission of the audience dimension from the 5W model, absence of longitudinal feedback measures, and demographic differences between the two practitioner groups. Robustness checks indicated that measured demographic differences did not fully explain the main findings. Future research should expand the geographic coverage of Wuqinxi samples, incorporate objective behavioral metrics and digital trace measures, and use audience-centered qualitative designs to examine how different population groups interpret, access, and sustain Wuqinxi participation. Longitudinal designs and more demographically balanced sampling would also help address the study’s cross-sectional constraints and group differences.

## Data Availability

The original contributions presented in the study are included in the article/Supplementary material, further inquiries can be directed to the corresponding author.
