# Supplementary material for: Propagating wellness: understanding the communication mechanisms of yoga and Wuqinxi (five animal frolics) via Lasswell’s 5W framework
Source: Front Public Health. 2026 Jul 23;14:1850942. doi: 10.3389/fpubh.2026.1850942 (PMC13443314; doi:10.3389/fpubh.2026.1850942)
Supplement: Supplementary file 1 [file Table_1.docx]

**Supplementary Materials Overview**

These supplementary materials support the findings reported in the manuscript entitled “*Propagating Wellness: Understanding the Communication Mechanisms of Yoga and Wuqinxi in China via Lasswell’s 5W Framework*”. They contain the supplementary tables, survey instruments, anonymized response data, and analysis code used in the study. The contents are organized as follows:

**File 1: Supplementary_Tables_S1-S16.docx**

This document contains all supplementary tables (S1–S16) referenced in the manuscript, including detailed regression results for overall effects, mediation effects, moderation effects, and robustness checks.

**Files 2–5:** The questionnaire and corresponding response data for the yoga and Wuqinxi studies. Each questionnaire originally contained 50 items; only the 27 validated questions used in the final analysis are retained to ensure data quality and relevance to our research objectives.

**Files 6–13:** The original Jupyter Notebooks (.ipynb) for hypothesis testing. For ease of review, HTML versions of each notebook are also provided.

**File 14:** Scientific research ethics review certificate confirming that this low-risk study followed standard ethical protocols. All participants provided informed consent, and the data were collected anonymously.

These materials are provided to support the review process. Thank you for your time and consideration.

Best regards,

Yong Song

Anqing Normal University

April 9, 2026
