# Supplementary material for: Propagating wellness: understanding the communication mechanisms of yoga and Wuqinxi (five animal frolics) via Lasswell’s 5W framework
Source: Front Public Health. 2026 Jul 23;14:1850942. doi: 10.3389/fpubh.2026.1850942 (PMC13443314; doi:10.3389/fpubh.2026.1850942)
Supplement: Supplementary file 2 [file Data_sheet_1.zip › Supplementary_Materials/13Testing_for_Communication_Channels.html]

传播渠道检验0606


In [1]:

```
import pandas as pd
import warnings
warnings.filterwarnings('ignore')
```

In [2]:

```
import os
import pandas as pd
dir_=r"C:\Users\admin\Desktop\1128第八次课"
os.chdir(dir_)
```

In [3]:

```
data1=pd.read_excel("五禽戏答卷(1).xlsx")
data2=pd.read_excel("瑜伽答卷(1).xlsx")
data1['type']=0
data2['type']=1
data2.columns = data1.columns
```

In [4]:

```
data = pd.concat([data1,data2])
```

In [5]:

```
data.columns
```

Out[5]:

```
Index(['序号', '所用时间', '来自IP', '1、您的性别是           。', '2、您的年龄是            。',
       '3、您的职业是            。', '4、您所在会馆的练习者人数为           。', '5、您练习该项目的时间有多长?',
       '6、您所在会馆的授课时间是         。', '7、您练习该项目是为了健康类目的，例如强身健体。',
       '8、您练习该项目是为了治疗类目的，例如产后恢复、治疗肩颈、脊柱侧弯等疾病。', '9、您练习该项目是出自对美与时尚的追求，例如减脂塑形。',
       '10、您练习该项目是为了促进社会交往，例如丰富业余生活。', '11、您练习该项目是为了缓解压力与改善心情。',
       '12、您认为观看该项目是美的享受。', '13、您认为练习该项目是美的体验。', '14、您认为您所在会馆提供了丰富多样的课程。',
       '15、您对您所在会馆提供的课程感到满意。', '16、您所在会馆的上课形式包括大班课（大于等于20人）。',
       '17、您所在会馆的上课形式包括小班课（10人以内）。', '18、您所在会馆的上课形式包括一对一私教课。',
       '19、您购买了与该项目相关的基本产品（如练功服）。', '20、您购买了与该项目相关的进阶产品（如练习器材等）。',
       '21、您购买了与该项目相关的指导产品（如书籍、视频资料等）。', '22、您没有购买与该项目相关的任何产品。',
       '23、您所在会馆的教练员接受过专业培训。', '24、您所在会馆的教练员拥有教练资格证书。',
       '25、您所在会馆的教练员拥有5年以上教学经验。', '26、您所在会馆的教练员是以教练为职业的专业工作者。',
       '27、您所在会馆的教练员定期进行培训、考核。', '28、您所在会馆的教学内容包括技术动作。',
       '29、您所在会馆的技术动作教学是多样的，根据不同的教学目标进行针对性的组合设计，不是固定套路的。',
       '30、您所在会馆的教学内容包括每一式的功理、功效、特点。', '31、您所在会馆的教学内容包括呼吸、意念。',
       '32、您所在会馆的教学内容包括除上述（技术动作，每一式的功理、功效与特点，呼吸，意念）外的其他内容。',
       '33、练习该项目，您感到自己的身体素质得到了显著提升（强身健体）。', '34、练习该项目丰富了您的业余生活。',
       '35、练习该项目帮助您扩大了社会交往圈。', '36、练习该项目满足了您对美与时尚的追求。',
       '37、练习该项目有效地缓解了您的压力，并改善了您的心情。', '38、您所在会馆的经费来源包括练习者交费。',
       '39、您所在会馆的经费来源包括企业资助。', '40、您所在会馆的经费来源包括政府资助。',
       '41、您所在会馆的经费来源除上述外，还包括其他来源。', '42、您对所在会馆的授课时间安排感到满意。',
       '43、您通过户外广告，了解到所在会馆的该项目。', '44、您通过互联网，了解到所在会馆的该项目。',
       '45、您通过书籍、报纸或杂志上的文章，了解到所在会馆的该项目。', '46、您通过观看相关比赛，了解到所在会馆的该项目。',
       '47、您通过朋友或熟人的口口相传，了解到了所在会馆的该项目。',
       '48、您所在会馆提供了丰富的基础设施配套（如更衣室、淋浴间、卫生间等）。',
       '49、您对所在会馆的基础设施（如更衣室、淋浴间、卫生间等）感到满意。', '50、您所在会馆重视会员对基础设施的反馈意见。',
       'type'],
      dtype='object')
```

In [6]:

```
import pandas as pd
import numpy as np
from factor_analyzer import FactorAnalyzer, calculate_bartlett_sphericity, calculate_kmo
import matplotlib.pyplot as plt
import seaborn as sns
```

In [7]:

```
columns = [ '15、您对您所在会馆提供的课程感到满意。','33、练习该项目，您感到自己的身体素质得到了显著提升（强身健体）。','34、练习该项目丰富了您的业余生活。', 
       '35、练习该项目帮助您扩大了社会交往圈。','36、练习该项目满足了您对美与时尚的追求。','37、练习该项目有效地缓解了您的压力，并改善了您的心情。'] 
df_dependent = data[columns]
```

In [8]:

```
#以上题项已通过因子检验，代表传播效果。
```

In [9]:

```
data['y']=df_dependent.mean(axis=1)
#对传播效果取平均值，赋值给data['y']
```

In [10]:

```
qudao = data[[  '44、您通过互联网，了解到所在会馆的该项目。']]
```

In [11]:

```
xiaofei = data[['19、您购买了与该项目相关的基本产品（如练功服）。', '20、您购买了与该项目相关的进阶产品（如练习器材等）。',
       '21、您购买了与该项目相关的指导产品（如书籍、视频资料等）。']]
```

In [12]:

```
#以上题项通过因子检验，代表练习者的消费行为。
```

In [13]:

```
data['传播渠道']=qudao.mean(axis=1)
df1 = data[['传播渠道','y']]
df1['中介_消费']=xiaofei.mean(axis=1)
df1['调节_类型']=data['type']
```

In [14]:

```
## 假设检验
import pandas as pd
import statsmodels.api as sm
from statsmodels.stats.mediation import Mediation
from statsmodels.formula.api import ols
```

In [15]:

```
# 主效应检验 Overall Effect of Channels Modernization on Communication Effectiveness
X = sm.add_constant(df1['传播渠道'])  # 添加常数项
y = df1['y']  # 被解释变量

# 运行回归模型
model_h1 = sm.OLS(y, X).fit()
print(model_h1.summary())
```

```
                            OLS Regression Results                            
==============================================================================
Dep. Variable:                      y   R-squared:                       0.160
Model:                            OLS   Adj. R-squared:                  0.158
Method:                 Least Squares   F-statistic:                     73.79
Date:                Fri, 06 Jun 2025   Prob (F-statistic):           2.16e-16
Time:                        10:33:19   Log-Likelihood:                -195.77
No. Observations:                 389   AIC:                             395.5
Df Residuals:                     387   BIC:                             403.5
Df Model:                           1                                         
Covariance Type:            nonrobust                                         
==============================================================================
                 coef    std err          t      P>|t|      [0.025      0.975]
------------------------------------------------------------------------------
const          3.5733      0.081     44.013      0.000       3.414       3.733
传播渠道           0.1793      0.021      8.590      0.000       0.138       0.220
==============================================================================
Omnibus:                        6.628   Durbin-Watson:                   1.795
Prob(Omnibus):                  0.036   Jarque-Bera (JB):                6.661
Skew:                          -0.257   Prob(JB):                       0.0358
Kurtosis:                       3.383   Cond. No.                         16.5
==============================================================================

Warnings:
[1] Standard Errors assume that the covariance matrix of the errors is correctly specified.
```

In [16]:

```
#说明互联网等现代传播渠道对传播效果有显著的正向影响。
```

In [17]:

```
#中介效应检验 Mediating Effect of Consumption between Channels Modernization and Communication Outcomes
```

In [18]:

```
#总效应路径 a: 互联网传播渠道 → y
X_a = sm.add_constant(df1['传播渠道'])
model_a = sm.OLS(y, X_a).fit()
print("路径 a（传播渠道 → y 总效应）:")
print(model_a.summary())
```

```
路径 a（传播渠道 → y 总效应）:
                            OLS Regression Results                            
==============================================================================
Dep. Variable:                      y   R-squared:                       0.160
Model:                            OLS   Adj. R-squared:                  0.158
Method:                 Least Squares   F-statistic:                     73.79
Date:                Fri, 06 Jun 2025   Prob (F-statistic):           2.16e-16
Time:                        10:33:53   Log-Likelihood:                -195.77
No. Observations:                 389   AIC:                             395.5
Df Residuals:                     387   BIC:                             403.5
Df Model:                           1                                         
Covariance Type:            nonrobust                                         
==============================================================================
                 coef    std err          t      P>|t|      [0.025      0.975]
------------------------------------------------------------------------------
const          3.5733      0.081     44.013      0.000       3.414       3.733
传播渠道           0.1793      0.021      8.590      0.000       0.138       0.220
==============================================================================
Omnibus:                        6.628   Durbin-Watson:                   1.795
Prob(Omnibus):                  0.036   Jarque-Bera (JB):                6.661
Skew:                          -0.257   Prob(JB):                       0.0358
Kurtosis:                       3.383   Cond. No.                         16.5
==============================================================================

Warnings:
[1] Standard Errors assume that the covariance matrix of the errors is correctly specified.
```

In [19]:

```
# 路径 b: 传播渠道 → 中介_消费
X_b = sm.add_constant(df1['传播渠道'])  # 自变量加常数
mediator = df1['中介_消费']          # 中介变量

model_b = sm.OLS(mediator, X_b).fit()
print("路径 b（传播渠道 → 中介_消费）:")
print(model_b.summary())
```

```
路径 b（传播渠道 → 中介_消费）:
                            OLS Regression Results                            
==============================================================================
Dep. Variable:                  中介_消费   R-squared:                       0.148
Model:                            OLS   Adj. R-squared:                  0.145
Method:                 Least Squares   F-statistic:                     67.04
Date:                Fri, 06 Jun 2025   Prob (F-statistic):           3.91e-15
Time:                        10:34:09   Log-Likelihood:                -347.15
No. Observations:                 389   AIC:                             698.3
Df Residuals:                     387   BIC:                             706.2
Df Model:                           1                                         
Covariance Type:            nonrobust                                         
==============================================================================
                 coef    std err          t      P>|t|      [0.025      0.975]
------------------------------------------------------------------------------
const          3.0675      0.120     25.603      0.000       2.832       3.303
传播渠道           0.2522      0.031      8.188      0.000       0.192       0.313
==============================================================================
Omnibus:                       20.842   Durbin-Watson:                   1.567
Prob(Omnibus):                  0.000   Jarque-Bera (JB):               23.510
Skew:                          -0.520   Prob(JB):                     7.85e-06
Kurtosis:                       3.607   Cond. No.                         16.5
==============================================================================

Warnings:
[1] Standard Errors assume that the covariance matrix of the errors is correctly specified.
```

In [20]:

```
#说明互联网等现代传播渠道对 “练习者的消费行为” 有显著的正向影响。
```

In [21]:

```
# 路径 c: 中介_消费 → y（控制传播渠道）
X_c = sm.add_constant(df1[['传播渠道', '中介_消费']])  # 自变量包含教练和中介变量
y = df1['y']  # 因变量

model_c = sm.OLS(y, X_c).fit()
print("路径 c（传播渠道 + 中介_消费 → y）:")
print(model_c.summary())
```

```
路径 c（传播渠道 + 中介_消费 → y）:
                            OLS Regression Results                            
==============================================================================
Dep. Variable:                      y   R-squared:                       0.384
Model:                            OLS   Adj. R-squared:                  0.381
Method:                 Least Squares   F-statistic:                     120.5
Date:                Fri, 06 Jun 2025   Prob (F-statistic):           2.18e-41
Time:                        10:34:31   Log-Likelihood:                -135.36
No. Observations:                 389   AIC:                             276.7
Df Residuals:                     386   BIC:                             288.6
Df Model:                           2                                         
Covariance Type:            nonrobust                                         
==============================================================================
                 coef    std err          t      P>|t|      [0.025      0.975]
------------------------------------------------------------------------------
const          2.4993      0.114     21.879      0.000       2.275       2.724
传播渠道           0.0910      0.019      4.695      0.000       0.053       0.129
中介_消费          0.3501      0.030     11.858      0.000       0.292       0.408
==============================================================================
Omnibus:                       13.693   Durbin-Watson:                   1.943
Prob(Omnibus):                  0.001   Jarque-Bera (JB):               28.956
Skew:                          -0.078   Prob(JB):                     5.16e-07
Kurtosis:                       4.327   Cond. No.                         38.0
==============================================================================

Warnings:
[1] Standard Errors assume that the covariance matrix of the errors is correctly specified.
```

In [22]:

```
#调节效应 Moderating Effect of Sports Type on Channels Modernization and Communication Outcomes
```

In [23]:

```
df1['传播渠道*调节_类型'] = df1['传播渠道'] * df1['调节_类型']
X_a_mod = sm.add_constant(df1[['传播渠道', '调节_类型', '传播渠道*调节_类型']])
mediator = df1['中介_消费']
model_a_mod = sm.OLS(mediator, X_a_mod).fit()
print("自变量->中介变量 路径 a 调节效应:")
print(model_a_mod.summary())
```

```
自变量->中介变量 路径 a 调节效应:
                            OLS Regression Results                            
==============================================================================
Dep. Variable:                  中介_消费   R-squared:                       0.315
Model:                            OLS   Adj. R-squared:                  0.310
Method:                 Least Squares   F-statistic:                     58.99
Date:                Fri, 06 Jun 2025   Prob (F-statistic):           2.12e-31
Time:                        10:34:54   Log-Likelihood:                -304.66
No. Observations:                 389   AIC:                             617.3
Df Residuals:                     385   BIC:                             633.2
Df Model:                           3                                         
Covariance Type:            nonrobust                                         
==============================================================================
                 coef    std err          t      P>|t|      [0.025      0.975]
------------------------------------------------------------------------------
const          2.2394      0.157     14.239      0.000       1.930       2.549
传播渠道           0.4255      0.042     10.028      0.000       0.342       0.509
调节_类型          1.7732      0.218      8.122      0.000       1.344       2.202
传播渠道*调节_类型    -0.3695      0.057     -6.528      0.000      -0.481      -0.258
==============================================================================
Omnibus:                       24.921   Durbin-Watson:                   1.839
Prob(Omnibus):                  0.000   Jarque-Bera (JB):               29.138
Skew:                          -0.574   Prob(JB):                     4.71e-07
Kurtosis:                       3.693   Cond. No.                         45.8
==============================================================================

Warnings:
[1] Standard Errors assume that the covariance matrix of the errors is correctly specified.
```

In [24]:

```
df1['传播渠道*调节_类型'] = df1['传播渠道'] * df1['调节_类型']
y = df1['y']
X_b_mod = sm.add_constant(df1[['传播渠道', '调节_类型', '传播渠道*调节_类型']])
model_b_mod = sm.OLS(y, X_b_mod).fit()
print("路径 b 调节效应:")
print(model_b_mod.summary())
```

```
路径 b 调节效应:
                            OLS Regression Results                            
==============================================================================
Dep. Variable:                      y   R-squared:                       0.269
Model:                            OLS   Adj. R-squared:                  0.263
Method:                 Least Squares   F-statistic:                     47.12
Date:                Fri, 06 Jun 2025   Prob (F-statistic):           5.83e-26
Time:                        10:35:08   Log-Likelihood:                -168.89
No. Observations:                 389   AIC:                             345.8
Df Residuals:                     385   BIC:                             361.6
Df Model:                           3                                         
Covariance Type:            nonrobust                                         
==============================================================================
                 coef    std err          t      P>|t|      [0.025      0.975]
------------------------------------------------------------------------------
const          2.9958      0.111     27.004      0.000       2.778       3.214
传播渠道           0.3223      0.030     10.770      0.000       0.263       0.381
调节_类型          1.1448      0.154      7.433      0.000       0.842       1.448
传播渠道*调节_类型    -0.2737      0.040     -6.854      0.000      -0.352      -0.195
==============================================================================
Omnibus:                        4.680   Durbin-Watson:                   1.873
Prob(Omnibus):                  0.096   Jarque-Bera (JB):                5.568
Skew:                          -0.104   Prob(JB):                       0.0618
Kurtosis:                       3.548   Cond. No.                         45.8
==============================================================================

Warnings:
[1] Standard Errors assume that the covariance matrix of the errors is correctly specified.
```

In [25]:

```
df1['中介_消费*调节_类型'] = df1['中介_消费'] * df1['调节_类型']
X_c_mod = sm.add_constant(df1[['传播渠道',  '调节_类型', '传播渠道*调节_类型','中介_消费','中介_消费*调节_类型']])
y = df1['y']
model_c_mod = sm.OLS(y, X_c_mod).fit()
print("X->中介变量->Y（调节作用在X->中介上，中介->Y）")
print(model_c_mod.summary())
```

```
X->中介变量->Y（调节作用在X->中介上，中介->Y）
                            OLS Regression Results                            
==============================================================================
Dep. Variable:                      y   R-squared:                       0.412
Model:                            OLS   Adj. R-squared:                  0.404
Method:                 Least Squares   F-statistic:                     53.60
Date:                Fri, 06 Jun 2025   Prob (F-statistic):           4.12e-42
Time:                        10:35:23   Log-Likelihood:                -126.55
No. Observations:                 389   AIC:                             265.1
Df Residuals:                     383   BIC:                             288.9
Df Model:                           5                                         
Covariance Type:            nonrobust                                         
===============================================================================
                  coef    std err          t      P>|t|      [0.025      0.975]
-------------------------------------------------------------------------------
const           2.2865      0.141     16.253      0.000       2.010       2.563
传播渠道            0.1875      0.033      5.708      0.000       0.123       0.252
调节_类型           0.6243      0.255      2.449      0.015       0.123       1.126
传播渠道*调节_类型     -0.1561      0.041     -3.841      0.000      -0.236      -0.076
中介_消费           0.3167      0.044      7.151      0.000       0.230       0.404
中介_消费*调节_类型    -0.0103      0.065     -0.159      0.874      -0.138       0.117
==============================================================================
Omnibus:                       13.845   Durbin-Watson:                   1.931
Prob(Omnibus):                  0.001   Jarque-Bera (JB):               30.484
Skew:                           0.017   Prob(JB):                     2.40e-07
Kurtosis:                       4.371   Cond. No.                         108.
==============================================================================

Warnings:
[1] Standard Errors assume that the covariance matrix of the errors is correctly specified.
```

In [ ]:
