# Supplementary material for: Propagating wellness: understanding the communication mechanisms of yoga and Wuqinxi (five animal frolics) via Lasswell’s 5W framework
Source: Front Public Health. 2026 Jul 23;14:1850942. doi: 10.3389/fpubh.2026.1850942 (PMC13443314; doi:10.3389/fpubh.2026.1850942)
Supplement: Supplementary file 2 [file Data_sheet_1.zip › Supplementary_Materials/14Ethics_Statement_Anqing_Normal_University.pdf]

安慶師範大學
ANQING NORMAL UNIVERSITY

科学研究伦理审查证明

经审核，Yong Song 等人提交的研究方案符合伦理原则，相关信息如下：

<table>
<tr><td rowspan="2">研究名称
Research Title</td><td colspan="5">传播健康：基于拉斯韦尔5W 框架理解瑜伽与五禽戏在中国的传播机制</td></tr>
<tr><td colspan="5">Propagating Wellness: Understanding the Communication Mechanisms of Yoga and Wuqinxi in China via Lasswell's 5W Framework</td></tr>
<tr><td>申请人
Applicant</td><td>宋勇</td><td>职称/学位
Title/Degree</td><td>讲师/硕士</td><td>邮箱 Email</td><td>songyong@aqnu.edu.cn</td></tr>
<tr><td>所在院系(部门)
School/Department</td><td colspan="3">外国语学院</td><td>申请日期
Application date</td><td>2026年3月27日</td></tr>
<tr><td>研究类型
Type of Study</td><td colspan="3">社会调查（匿名问卷调查与非结构性访谈）</td><td>研究对象
Participants</td><td>瑜伽与五禽戏练习者</td></tr>
<tr><td>数据收集方式
Data Collection</td><td colspan="5">线上问卷平台与线下现场发放，所有数据匿名处理，仅用于学术研究</td></tr>
<tr><td>伦理审查意见
Results of inspection</td><td colspan="5">该研究仅涉及匿名问卷调查与访谈，不涉及个人敏感信息、不干预人身、不涉及弱势群体，属于低风险研究。根据我校科研伦理相关规定，无需提交伦理委员会进行正式审批。</td></tr>
</table>

安庆师范大学学术委员会

科学研究与学术道德（科学伦理）专门委员会

2026 年 3 月 27 日

Scientific Research Ethics Review Certificate

After review, the research protocol submitted by Yong Song and colleagues complies with ethical principles. The relevant information is as follows:

<table>
<tr><td>Research Title</td><td colspan="5">Propagating Wellness: Understanding the Communication Mechanisms of Yoga and Wuqinxi in China via Lasswell's 5W Framework</td></tr>
<tr><td>Applicant</td><td>Yong Song</td><td>Title/Degree</td><td>Lecturer / Master</td><td>Email</td><td>songyong@aqnu.edu.cn</td></tr>
<tr><td>School/Department</td><td colspan="3">School of Foreign Languages</td><td>Application Date</td><td>MAR. 27, 2026</td></tr>
<tr><td>Type of Study</td><td colspan="3">Social Survey
(anonymous questionnaire and semi-structured interviews)</td><td>Participants</td><td>Yoga and Wuqinxi practitioners</td></tr>
<tr><td>Data Collection</td><td colspan="5">Online survey platforms and on-site distribution. All data were anonymized and used solely for academic research.</td></tr>
<tr><td>Results of Inspection</td><td colspan="5">This study involves only anonymous questionnaires and interviews, does not involve sensitive personal information, medical interventions, or vulnerable populations, and is classified as low risk. In accordance with the institutional regulations on research ethics, formal approval from the ethics committee is not required.</td></tr>
</table>

Academic Committee of Anqing Normal University

Subcommittee for Scientific Research and Academic Ethics

March 27, 2026
