# Supplementary material for: Propagating wellness: understanding the communication mechanisms of yoga and Wuqinxi (five animal frolics) via Lasswell’s 5W framework
Source: Front Public Health. 2026 Jul 23;14:1850942. doi: 10.3389/fpubh.2026.1850942 (PMC13443314; doi:10.3389/fpubh.2026.1850942)
Supplement: Supplementary file 2 [file Data_sheet_1.zip › Supplementary_Materials/1Supplementary_Tables_S1-S16.docx]

**Table S1** Overall Effect of Instructor Professionalism on Communication Effectiveness

|  | Non-standardized coefficient (B) | Standard error | t-value | Significance | [0.025 0.975] |
| --- | --- | --- | --- | --- | --- |
| Constants | 2.0907 | 0.106 | 19.733 | 0.000 | 1.882 2.299 |
| the Level of Professionalism among Instructor | 0.5222 | 0.025 | 20.583 | 0.000 | 0.472 0.572 |

**Table S2** Overall Effect of Venue Facilities Completeness on Communication Effectiveness

|  | Non-standardized Coefficient (B) | Standard Error | t-value | Significance | [0.025 0.975] |
| --- | --- | --- | --- | --- | --- |
| Constants | 2.7195 | 0.111 | 24.400 | 0.000 | 2.500 2.939 |
| Venue Facilities | 0.3706 | 0.027 | 13.903 | 0.000 | 0.318 0.423 |

**Table S3** Overall Effect of Content Diversity on Communication Effectiveness

|  | Non-standardized coefficient (B) | Standard error | t-value | Significance | [0.025 0.975] |
| --- | --- | --- | --- | --- | --- |
| Constants | 1.5708*** | 0.137 | 11.454 | 0.000 | 1.301 1.840 |
| Diversity of  Disseminated Content | 0.6464*** | 0.033 | 19.655 | 0.000 | 0.582 0.711 |

**Table S4** Overall Effect of Channels Modernization on Communication Effectiveness

|  | Non-standardized Coefficient (B) | Standard Error | t-value | Significance | [0.025 0.975] |
| --- | --- | --- | --- | --- | --- |
| Constants | 3.5733 | 0.081 | 44.013 | 0.000 | 3.414 3.733 |
| Modernization of Communication Channels | 0.1793 | 0.021 | 8.590 | 0.000 | 0.138 0.220 |

**Table S5** Mediating Effect of Consumption between Instructor Professionalism and Communication Outcomes

|  | Dependent Variable: Communication Effectiveness | | | Dependent Variable: Consumption | | | Dependent Variable: Communication Effectiveness | | |
| --- | --- | --- | --- | --- | --- | --- | --- | --- | --- |
| Variables | B | SE | t | B | SE | t | B | SE | t |
| Constants | 2.0907*** | 0.106 | 19.733 | 1.1738*** | 0.171 | 6.879 | 1.9229*** | 0.109 | 17.583 |
| Instructor | 0.5222*** | 0.025 | 20.583 | 0.6881*** | 0.041 | 16.840 | 0.4238*** | 0.033 | 13.025 |
| Consumption |  |  |  |  |  |  | 0.1429*** | 0.031 | 4.648 |
| R2 | 0.523 | | | 0.423 | | | 0.548 | | |
| F | 423.7*** | | | 283.6*** | | | 233.9*** | | |

Note: *p＜0.05，**p＜0.01，***p＜0.001

**Table S6** Mediating Effect of Consumption between Venue Facilities Completeness and Communication Outcomes

|  | Dependent Variable: Communication Effectiveness | | | Dependent variable: Consumption | | | Dependent Variable: Communication Effectiveness | | |
| --- | --- | --- | --- | --- | --- | --- | --- | --- | --- |
| Variables | B | SE | t | B | SE | t | B | SE | t |
| Constants | 2.7195*** | 0.111 | 24.400 | 1.6466*** | 0.158 | 10.402 | 2.2920*** | 0.117 | 19.532 |
| Facilities | 0.3706*** | 0.027 | 13.903 | 0.5747*** | 0.038 | 15.178 | 0.2215*** | 0.031 | 7.066 |
| Consumption |  |  |  |  |  |  | 0.2596*** | 0.033 | 7.793 |
| R2 | 0.333 | | | 0.373 | | | 0.424 | | |
| F | 193.3*** | | | 230.4*** | | | 141.9*** | | |

Note: *p＜0.05，**p＜0.01，***p＜0.001

**Table S7** Mediating Effect of Consumption between Content Diversity and Communication Outcomes

|  | Dependent Variable: Communication Effectiveness | | | Dependent Variable: Consumption | | | Dependent Variable: Communication Effectiveness | | |
| --- | --- | --- | --- | --- | --- | --- | --- | --- | --- |
| Variables | B | SE | t | B | SE | t | B | SE | t |
| Constants | 1.5708*** | 0.137 | 11.454 | 0.4765* | 0.219 | 2.179 | 1.4939*** | 0.134 | 11.189 |
| Communication  Content | 0.6464*** | 0.033 | 19.655 | 0.8547*** | 0.052 | 16.299 | 0.5086*** | 0.041 | 12.307 |
| Practitioners' Consumption Behavior |  |  |  |  |  |  | 0.1612*** | 0.031 | 5.226 |
| R^2^ | 0.500 | | | 0.407 | | | 0.533 | | |
| F | 386.3*** | | | 265.6*** | | | 219.9*** | | |

Note: *p＜0.05，**p＜0.01，***p＜0.001

**Table S8** Mediating Effect of Consumption between Channels Modernization and Communication Outcomes

|  | Dependent Variable: Communication Effectiveness | | | Dependent Variable: Consumption | | | Dependent Variable: Communication Effectiveness | | |
| --- | --- | --- | --- | --- | --- | --- | --- | --- | --- |
| Variables | B | SE | t | B | SE | t | B | SE | t |
| Constants | 3.5733*** | 0.081 | 44.013 | 3.0675*** | 0.120 | 25.603 | 2.4993*** | 0.114 | 21.879 |
| Channels | 0.1793*** | 0.021 | 8.590 | 0.2522*** | 0.031 | 8.188 | 0.0910*** | 0.019 | 4.695 |
| Consumption |  |  |  |  |  |  | 0.3501*** | 0.030 | 11.858 |
| R^2^ | 0.160 | | | 0.148 | | | 0.384 | | |
| F | 73.79*** | | | 67.04*** | | | 120.5*** | | |

Note: *p＜0.05，**p＜0.01，***p＜0.001

**Table S9** Moderating Effect of Sports Type on Instructor Professionalism and Communication Outcomes

|  | Dependent Variable: Communication Effectiveness | | | Dependent Variable: Consumption | | | Dependent Variable: Communication Effectiveness | | |
| --- | --- | --- | --- | --- | --- | --- | --- | --- | --- |
| Variables | B | SE | t | B | SE | t | B | SE | t |
| Constants | 1.9238*** | 0.121 | 15.891 | 1.2092*** | 0.193 | 6.273 | 1.8242*** | 0.128 | 14.248 |
| Instructor | 0.5699*** | 0.030 | 18.728 | 0.6538*** | 0.048 | 13.495 | 0.5160*** | 0.041 | 12.658 |
| Sports  Type | 0.6376 | 0.273 | 2.338 | 0.6483 | 0.434 | 1.493 | 0.3149 | 0.281 | 1.122 |
| Instructor*  Sports Type | -0.1605 | 0.064 | -2.506 | -0.1045 | 0.102 | -1.025 | -0.2315** | 0.072 | -3.207 |
| Consumption |  |  |  |  |  |  | 0.0824 | 0.043 | 1.905 |
| Consumption*  Sports Type |  |  |  |  |  |  | 0.1450* | 0.062 | 2.345 |
| R2 | 0.532 | | | 0.447 | | | 0.566 | | |
| F | 146.0*** | | | 103.9*** | | | 100.0*** | | |

Note: *p＜0.05，**p＜0.01，***p＜0.001

**Table S10** Moderating Effect of Sports Type on Adequacy of Venue Facilities and Communication Outcomes

|  | Dependent Variable: Communication Effectiveness | | | Dependent Variable: Consumption | | | Dependent Variable: Communication Effectiveness | | |
| --- | --- | --- | --- | --- | --- | --- | --- | --- | --- |
| Variables | B | SE | t | B | SE | t | B | SE | t |
| Constants | 2.6480*** | 0.130 | 20.411 | 1.7857*** | 0.182 | 9.806 | 2.1223*** | 0.144 | 14.728 |
| Venues | 0.3928*** | 0.033 | 11.845 | 0.5171*** | 0.047 | 11.110 | 0.2405*** | 0.038 | 6.266 |
| Sports  Type | 0.2244 | 0.299 | 0.752 | 0.0952 | 0.419 | 0.227 | 0.2975 | 0.303 | 0.980 |
| Venue  *Sport Type | -0.0596 | 0.070 | -0.857 | 0.0199 | 0.098 | 0.204 | -0.0366 | 0.074 | -0.496 |
| Consumption |  |  |  |  |  |  | 0.2944*** | 0.045 | 6.612 |
| Consumption  *Sport Type |  |  |  |  |  |  | -0.0538 | 0.068 | -0.791 |
| R^2^ | 0.335 | | | 0.390 | | | 0.433 | | |
| F | 64.73*** | | | 81.94*** | | | 58.41*** | | |

Note: *p＜0.05，**p＜0.01，***p＜0.001

**Table S11** Moderating Effect of Sports Type on Diversity of Content and Communication Outcomes

|  | Dependent variable: Communication Effectiveness | | | Dependent variable: Consumption | | | Dependent variable: Communication effectiveness | | |
| --- | --- | --- | --- | --- | --- | --- | --- | --- | --- |
| Variables | B | SE | t | B | SE | t | B | SE | t |
| Constants | 1.4099*** | 0.155 | 9.107 | 0.5328* | 0.239 | 2.227 | 1.3601*** | 0.151 | 9.001 |
| Content | 0.6831*** | 0.038 | 17.909 | 0.8053*** | 0.059 | 13.664 | 0.6079*** | 0.051 | 11.831 |
| Sports  Type | 0.8713* | 0.345 | 2.523 | 0.7321 | 0.534 | 1.372 | 0.6228 | 0.339 | 1.839 |
| Content*  Sports Type | -0.2010* | 0.082 | -2.456 | -0.1076 | 0.126 | -0.851 | -0.2903** | 0.092 | -3.146 |
| Consumption |  |  |  |  |  |  | 0.0934* | 0.045 | 2.095 |
| Consumption*  Sports Type |  |  |  |  |  |  | 0.1424* | 0.064 | 2.239 |
| R^2^ | 0.508 | | | 0.453 | | | 0.545 | | |
| F | 132.6*** | | | 106.1*** | | | 91.91*** | | |

Note: *p＜0.05，**p＜0.01，***p＜0.001

**Table S12** Moderating Effect of Sports Type on Channels Modernization and Communication Outcomes

|  | Dependent variable: Communication Effectiveness | | | Dependent Variable: Consumption | | | Dependent variable: Communication Effectiveness | | |
| --- | --- | --- | --- | --- | --- | --- | --- | --- | --- |
| Variables | B | SE | t | B | SE | t | B | SE | t |
| Constants | 2.9958*** | 0.111 | 27.004 | 2.2394*** | 0.157 | 14.239 | 2.2865*** | 0.141 | 16.253 |
| Channels | 0.3223*** | 0.030 | 10.770 | 0.4255*** | 0.042 | 10.028 | 0.1875*** | 0.033 | 5.708 |
| Sports  Type | 1.1448*** | 0.154 | 7.433 | 1.7732*** | 0.218 | 8.122 | 0.6243* | 0.255 | 2.449 |
| Channel*  Sports Type | -0.2737*** | 0.040 | -6.854 | -0.3695*** | 0.057 | -6.528 | -0.1561*** | 0.041 | -3.841 |
| Consumption |  |  |  |  |  |  | 0.3167*** | 0.044 | 7.151 |
| Consumption*Sports Type |  |  |  |  |  |  | -0.0103 | 0.065 | -0.159 |
| R^2^ | 0.269 | | | 0.315 | | | 0.412 | | |
| F | 47.12*** | | | 58.99*** | | | 53.60*** | | |

Note: *p＜0.05，**p＜0.01，***p＜0.001

**Table S13** Robustness Check of Instructor Professionalism on Communication Effectiveness

| Model Type | Coefficient | p-value | Significance (α=0.05) | R² | Sample Size |
| --- | --- | --- | --- | --- | --- |
| Main Effect Test | **0.5222** | **< 0.001** | **√** | **0.5226** | **389** |
| Main Effect Regression Model | **0.5207** | **< 0.001** | **√** | **0.5240** | **389** |
| Robustness Test | **0.5170** | **< 0.001** | **√** | **0.5247** | **389** |

**Table S14** Robustness Check of Venue Facilities Completeness on Communication Effectiveness

| Model Type | Coefficient | p-value | Significance (α=0.05) | R² | Sample Size |
| --- | --- | --- | --- | --- | --- |
| Main Effect Test | **0.3706** | **< 0.001** | **√** | **0.3331** | **389** |
| Main Effect Regression Model | **0.3953** | **< 0.001** | **√** | **0.3746** | **389** |
| Robustness Test | **0.3902** | **< 0.001** | **√** | **0.3760** | **389** |

**Table S15** Robustness Check of Content Diversity on Communication Effectiveness

| Model Type | Coefficient | p-value | Significance (α=0.05) | R² | Sample Size |
| --- | --- | --- | --- | --- | --- |
| Main Effect Test | **0.6464** | **< 0.001** | **√** | **0.4996** | **389** |
| Main Effect Regression Model | **0.6439** | **< 0.001** | **√** | **0.5034** | **389** |
| Robustness Test | **0.6357** | **< 0.001** | **√** | **0.5080** | **389** |

**Table S16** Robustness Check of Channels Modernization on Communication Effectiveness

| Model Type | Coefficient | p-value | Significance (α=0.05) | R² | Sample Size |
| --- | --- | --- | --- | --- | --- |
| Main Effect Test | **0.1793** | **< 0.001** | **√** | **0.1601** | **389** |
| Main Effect Regression Model | **0.1860** | **< 0.001** | **√** | **0.1782** | **389** |
| Robustness Test | **0.1799** | **< 0.001** | **√** | **0.1908** | **389** |
