# Supplementary material for: Propagating wellness: understanding the communication mechanisms of yoga and Wuqinxi (five animal frolics) via Lasswell’s 5W framework
Source: Front Public Health. 2026 Jul 23;14:1850942. doi: 10.3389/fpubh.2026.1850942 (PMC13443314; doi:10.3389/fpubh.2026.1850942)
Supplement: Supplementary file 2 [file Data_sheet_1.zip › Supplementary_Materials/4Wuqinxi_Questionnaire.docx]

**五禽戏练习者调查问卷**

尊敬的受访者

您好！

这是一份关于五禽戏练习者的调查问卷，如果您正在练习五禽戏，请填写此问卷。

请根据您个人的真实情况以及感受，选择真实答案以及最符合您态度的选项。您的回答对我们非常重要，没有正确或错误的答案，我们重视您的真实意见。

为便于统计，问卷中“该项目”指五禽戏。

本问卷共50题，请耐心填写，感谢您的支持！

1. 您的性别是 。

A.男性

B.女性

2. 您的年龄是	。

A.15岁及以下

B.16-25岁

C.26-35岁

D.36-45岁

E.46-55岁

F.56-65岁

G.66岁及以上

3. 您的职业是	。

A.政府工作人员

B.企业员工

C.医务工作者

D.教育工作者

E.商务工作者

F.农民

G.在校学生

H.退休人员

I.其他

4. 您所在会馆的练习者人数为	。

A. 小于50人

B. 50-99人

C. 100-200人

D. 大于200人

5. 您练习该项目的时间有多长？

A.之前练习过，现在中断了

B. ≤6个月

C.6-12个月

D. 1-3年

E. 3年以上

6. 您所在会馆的授课时间是	。

A. 每天上午，6:30-9:30左右

B. 每天下午，15:30-17:30左右

C. 每天晚上，19:00-21:00左右

D. 上午、下午、晚上均有授课

7. 您练习该项目是为了健康类目的，例如强身健体。

A.	非常不同意

B.	不同意

C.	中立

D.	同意

E.	非常同意

8. 您练习该项目是为了治疗类目的，例如产后恢复、治疗肩颈、脊柱侧弯等疾病。

A.	非常不同意

B.	不同意

C.	中立

D.	同意

E.	非常同意

9. 您练习该项目是出自对美与时尚的追求，例如减脂塑形。

A.	非常不同意

B.	不同意

C.	中立

D.	同意

E.	非常同意

10. 您练习该项目是为了促进社会交往，例如丰富业余生活。

A.	非常不同意

B.	不同意

C.	中立

D.	同意

E.	非常同意

11. 您练习该项目是为了缓解压力与改善心情。

A.	非常不同意

B.	不同意

C.	中立

D.	同意

E.	非常同意

12. 您认为观看该项目是美的享受。

A.	非常不同意

B.	不同意

C.	中立

D.	同意

E.	非常同意

13. 您认为练习该项目是美的体验。

A.	非常不同意

B.	不同意

C.	中立

D.	同意

E.	非常同意

14. 您认为您所在会馆提供了丰富多样的课程。

A.	非常不同意

B.	不同意

C.	中立

D.	同意

E.	非常同意

15. 您对您所在会馆提供的课程感到满意。

A.	非常不同意

B.	不同意

C.	中立

D.	同意

E.	非常同意

16. 您所在会馆的上课形式包括大班课（大于等于20人）。

A.	非常不同意

B.	不同意

C.	中立

D.	同意

E.	非常同意

17. 您所在会馆的上课形式包括小班课（10人以内）。

A.	非常不同意

B.	不同意

C.	中立

D.	同意

E.	非常同意

18. 您所在会馆的上课形式包括一对一私教课。

A.	非常不同意

B.	不同意

C.	中立

D.	同意

E.	非常同意

19. 您购买了与该项目相关的基本产品（如练功服）。

A.	非常不同意

B.	不同意

C.	中立

D.	同意

E.	非常同意

20. 您购买了与该项目相关的进阶产品（如练习器材等）。

A.	非常不同意

B.	不同意

C.	中立

D.	同意

E.	非常同意

21. 您购买了与该项目相关的指导产品（如书籍、视频资料等）。

A.	非常不同意

B.	不同意

C.	中立

D.	同意

E.	非常同意

22. 您没有购买与该项目相关的任何产品。

A.	非常不同意

B.	不同意

C.	中立

D.	同意

E.	非常同意

23. 您所在会馆的教练员接受过专业培训。

A.	非常不同意

B.	不同意

C.	中立

D.	同意

E.	非常同意

24. 您所在会馆的教练员拥有教练资格证书。

A.	非常不同意

B.	不同意

C.	中立

D.	同意

E.	非常同意

25. 您所在会馆的教练员拥有5年以上教学经验。

A.	非常不同意

B.	不同意

C.	中立

D.	同意

E.	非常同意

26. 您所在会馆的教练员是以教练为职业的专业工作者。

A.	非常不同意

B.	不同意

C.	中立

D.	同意

E.	非常同意

27. 您所在会馆的教练员定期进行培训、考核。

A.	非常不同意

B.	不同意

C.	中立

D.	同意

E.	非常同意

28. 您所在会馆的教学内容包括技术动作。

A.	非常不同意

B.	不同意

C.	中立

D.	同意

E.	非常同意

29. 您所在会馆的技术动作教学是多样的，根据不同的教学目标进行针对性的组合设计，不是固定套路的。

A.	非常不同意

B.	不同意

C.	中立

D.	同意

E.	非常同意

30. 您所在会馆的教学内容包括每一式的功理、功效、特点。

A.	非常不同意

B.	不同意

C.	中立

D.	同意

E.	非常同意

31. 您所在会馆的教学内容包括呼吸、意念。

A.	非常不同意

B.	不同意

C.	中立

D.	同意

E.	非常同意

32. 您所在会馆的教学内容包括除上述（技术动作，每一式的功理、功效与特点，呼吸，意念）外的其他内容。

A.	非常不同意

B.	不同意

C.	中立

D.	同意

E.	非常同意

33. 练习该项目，您感到自己的身体素质得到了显著提升（强身健体）。

A.	非常不同意

B.	不同意

C.	中立

D.	同意

E.	非常同意

34. 练习该项目丰富了您的业余生活。

A.	非常不同意

B.	不同意

C.	中立

D.	同意

E.	非常同意

35. 练习该项目帮助您扩大了社会交往圈。

A.	非常不同意

B.	不同意

C.	中立

D.	同意

E.	非常同意

36. 练习该项目满足了您对美与时尚的追求。

A.	非常不同意

B.	不同意

C.	中立

D.	同意

E.	非常同意

37. 练习该项目有效地缓解了您的压力，并改善了您的心情。

A.	非常不同意

B.	不同意

C.	中立

D.	同意

E.	非常同意

38. 您所在会馆的经费来源包括练习者交费。

A.	非常不同意

B.	不同意

C.	中立

D.	同意

E.	非常同意

39. 您所在会馆的经费来源包括企业资助。

A.	非常不同意

B.	不同意

C.	中立

D.	同意

E.	非常同意

40. 您所在会馆的经费来源包括政府资助。

A.	非常不同意

B.	不同意

C.	中立

D.	同意

E.	非常同意

41. 您所在会馆的经费来源除上述外，还包括其他来源。

A.	非常不同意

B.	不同意

C.	中立

D.	同意

E.	非常同意

42. 您对所在会馆的授课时间安排感到满意。

A.	非常不同意

B.	不同意

C.	中立

D.	同意

E.	非常同意

43. 您通过户外广告，了解到所在会馆的该项目。

A.	非常不同意

B.	不同意

C.	中立

D.	同意

E.	非常同意

44. 您通过互联网，了解到所在会馆的该项目。

A.	非常不同意

B.	不同意

C.	中立

D.	同意

E.	非常同意

45. 您通过书籍、报纸或杂志上的文章，了解到所在会馆的该项目。

A.	非常不同意

B.	不同意

C.	中立

D.	同意

E.	非常同意

46. 您通过观看相关比赛，了解到所在会馆的该项目。

A.	非常不同意

B.	不同意

C.	中立

D.	同意

E.	非常同意

47. 您通过朋友或熟人的口口相传，了解到了所在会馆的该项目。

A.	非常不同意

B.	不同意

C.	中立

D.	同意

E.	非常同意

48. 您所在会馆提供了丰富的基础设施配套（如更衣室、淋浴间、卫生间等）。

A.	非常不同意

B.	不同意

C.	中立

D.	同意

E.	非常同意

49. 您对所在会馆的基础设施（如更衣室、淋浴间、卫生间等）感到满意。

A.	非常不同意

B.	不同意

C.	中立

D.	同意

E.	非常同意

50. 您所在会馆重视会员对基础设施的反馈意见。

A.	非常不同意

B.	不同意

C.	中立

D.	同意

E.	非常同意
