# Supplementary material for: Propagating wellness: understanding the communication mechanisms of yoga and Wuqinxi (five animal frolics) via Lasswell’s 5W framework
Source: Front Public Health. 2026 Jul 23;14:1850942. doi: 10.3389/fpubh.2026.1850942 (PMC13443314; doi:10.3389/fpubh.2026.1850942)
Supplement: Supplementary file 2 [file Data_sheet_1.zip › Supplementary_Materials/7Testing_for_ Communicator.html]

传播者检验0604


In [1]:

```
import pandas as pd
import warnings
warnings.filterwarnings('ignore')
```

In [2]:

```
import os
import pandas as pd
dir_=r"C:\Users\admin\Desktop\1128第八次课"
os.chdir(dir_)
```

In [3]:

```
data1=pd.read_excel("五禽戏答卷(1).xlsx")
data2=pd.read_excel("瑜伽答卷(1).xlsx")
data1['type']=0
data2['type']=1
data2.columns = data1.columns
```

In [4]:

```
data = pd.concat([data1,data2])
```

In [5]:

```
data.columns
```

Out[5]:

```
Index(['序号', '所用时间', '来自IP', '1、您的性别是           。', '2、您的年龄是            。',
       '3、您的职业是            。', '4、您所在会馆的练习者人数为           。', '5、您练习该项目的时间有多长?',
       '6、您所在会馆的授课时间是         。', '7、您练习该项目是为了健康类目的，例如强身健体。',
       '8、您练习该项目是为了治疗类目的，例如产后恢复、治疗肩颈、脊柱侧弯等疾病。', '9、您练习该项目是出自对美与时尚的追求，例如减脂塑形。',
       '10、您练习该项目是为了促进社会交往，例如丰富业余生活。', '11、您练习该项目是为了缓解压力与改善心情。',
       '12、您认为观看该项目是美的享受。', '13、您认为练习该项目是美的体验。', '14、您认为您所在会馆提供了丰富多样的课程。',
       '15、您对您所在会馆提供的课程感到满意。', '16、您所在会馆的上课形式包括大班课（大于等于20人）。',
       '17、您所在会馆的上课形式包括小班课（10人以内）。', '18、您所在会馆的上课形式包括一对一私教课。',
       '19、您购买了与该项目相关的基本产品（如练功服）。', '20、您购买了与该项目相关的进阶产品（如练习器材等）。',
       '21、您购买了与该项目相关的指导产品（如书籍、视频资料等）。', '22、您没有购买与该项目相关的任何产品。',
       '23、您所在会馆的教练员接受过专业培训。', '24、您所在会馆的教练员拥有教练资格证书。',
       '25、您所在会馆的教练员拥有5年以上教学经验。', '26、您所在会馆的教练员是以教练为职业的专业工作者。',
       '27、您所在会馆的教练员定期进行培训、考核。', '28、您所在会馆的教学内容包括技术动作。',
       '29、您所在会馆的技术动作教学是多样的，根据不同的教学目标进行针对性的组合设计，不是固定套路的。',
       '30、您所在会馆的教学内容包括每一式的功理、功效、特点。', '31、您所在会馆的教学内容包括呼吸、意念。',
       '32、您所在会馆的教学内容包括除上述（技术动作，每一式的功理、功效与特点，呼吸，意念）外的其他内容。',
       '33、练习该项目，您感到自己的身体素质得到了显著提升（强身健体）。', '34、练习该项目丰富了您的业余生活。',
       '35、练习该项目帮助您扩大了社会交往圈。', '36、练习该项目满足了您对美与时尚的追求。',
       '37、练习该项目有效地缓解了您的压力，并改善了您的心情。', '38、您所在会馆的经费来源包括练习者交费。',
       '39、您所在会馆的经费来源包括企业资助。', '40、您所在会馆的经费来源包括政府资助。',
       '41、您所在会馆的经费来源除上述外，还包括其他来源。', '42、您对所在会馆的授课时间安排感到满意。',
       '43、您通过户外广告，了解到所在会馆的该项目。', '44、您通过互联网，了解到所在会馆的该项目。',
       '45、您通过书籍、报纸或杂志上的文章，了解到所在会馆的该项目。', '46、您通过观看相关比赛，了解到所在会馆的该项目。',
       '47、您通过朋友或熟人的口口相传，了解到了所在会馆的该项目。',
       '48、您所在会馆提供了丰富的基础设施配套（如更衣室、淋浴间、卫生间等）。',
       '49、您对所在会馆的基础设施（如更衣室、淋浴间、卫生间等）感到满意。', '50、您所在会馆重视会员对基础设施的反馈意见。',
       'type'],
      dtype='object')
```

In [6]:

```
import pandas as pd
import numpy as np
from factor_analyzer import FactorAnalyzer, calculate_bartlett_sphericity, calculate_kmo
import matplotlib.pyplot as plt
import seaborn as sns
```

In [11]:

```
columns = [ '15、您对您所在会馆提供的课程感到满意。','33、练习该项目，您感到自己的身体素质得到了显著提升（强身健体）。','34、练习该项目丰富了您的业余生活。', 
       '35、练习该项目帮助您扩大了社会交往圈。','36、练习该项目满足了您对美与时尚的追求。','37、练习该项目有效地缓解了您的压力，并改善了您的心情。'] 
df_dependent = data[columns]
```

In [12]:

```
# 初步因素分析
fa = FactorAnalyzer(rotation=None)
fa.fit(df_dependent)
```

Out[12]:

```
FactorAnalyzer(bounds=(0.005, 1), impute='median', is_corr_matrix=False,
        method='minres', n_factors=3, rotation=None, rotation_kwargs={},
        svd_method='randomized', use_smc=True)
```

In [13]:

```
ev, v = fa.get_eigenvalues()
# 查看方差解释率
variance = fa.get_factor_variance()

# 查看方差解释率
variance = fa.get_factor_variance()
# 获取实际因素数量
num_factors = min(len(ev), len(variance[1]), len(variance[2]))

# 构建表格
result_table = pd.DataFrame({
    '组件': [str(i + 1) for i in range(num_factors)],  # 动态生成组件编号
    '初始特征值_总计': ev[:num_factors],
    '初始特征值_方差百分比': [round(v * 100, 3) for v in variance[1][:num_factors]],
    '初始特征值_累积百分比': [round(v * 100, 3) for v in variance[2][:num_factors]],
    '提取载荷平方和_总计': ev[:num_factors],
    '提取载荷平方和_方差百分比': [round(v * 100, 3) for v in variance[1][:num_factors]],
    '提取载荷平方和_累积百分比': [round(v * 100, 3) for v in variance[2][:num_factors]],
})
```

In [14]:

```
result_table
```

Out[14]:

|  | 组件 | 初始特征值\_总计 | 初始特征值\_方差百分比 | 初始特征值\_累积百分比 | 提取载荷平方和\_总计 | 提取载荷平方和\_方差百分比 | 提取载荷平方和\_累积百分比 |
| --- | --- | --- | --- | --- | --- | --- | --- |
| 0 | 1 | 2.820524 | 39.740 | 39.740 | 2.820524 | 39.740 | 39.740 |
| 1 | 2 | 0.752688 | 9.052 | 48.792 | 0.752688 | 9.052 | 48.792 |
| 2 | 3 | 0.709636 | 4.274 | 53.066 | 0.709636 | 4.274 | 53.066 |

In [15]:

```
#以上题项已通过因子检验，代表传播效果。
```

In [16]:

```
data['y']=df_dependent.mean(axis=1)
#对传播效果取平均值，赋值给data['y']
```

In [17]:

```
jiaolian = data[['23、您所在会馆的教练员接受过专业培训。', '24、您所在会馆的教练员拥有教练资格证书。',
       '25、您所在会馆的教练员拥有5年以上教学经验。', '26、您所在会馆的教练员是以教练为职业的专业工作者。',
       '27、您所在会馆的教练员定期进行培训、考核。']]
```

In [18]:

```
#以上题项已通过因子检验，代表教练员的专业化水平。
```

In [19]:

```
xiaofei = data[['19、您购买了与该项目相关的基本产品（如练功服）。', '20、您购买了与该项目相关的进阶产品（如练习器材等）。',
       '21、您购买了与该项目相关的指导产品（如书籍、视频资料等）。']]
```

In [20]:

```
#以上题项通过因子检验，代表练习者的消费行为。
```

In [21]:

```
data['教练']=jiaolian.mean(axis=1)
df1 = data[['教练','y']]
df1['中介_消费']=xiaofei.mean(axis=1)
df1['调节_类型']=data['type']
```

In [22]:

```
## 假设检验
import pandas as pd
import statsmodels.api as sm
from statsmodels.stats.mediation import Mediation
from statsmodels.formula.api import ols
```

In [23]:

```
# 主效应检验 Overall Effect of Instructor Professionalism on Communication Effectiveness
X = sm.add_constant(df1['教练'])  # 添加常数项
y = df1['y']  # 被解释变量

# 运行回归模型
model_h1 = sm.OLS(y, X).fit()
print(model_h1.summary())
```

```
                            OLS Regression Results                            
==============================================================================
Dep. Variable:                      y   R-squared:                       0.523
Model:                            OLS   Adj. R-squared:                  0.521
Method:                 Least Squares   F-statistic:                     423.7
Date:                Wed, 04 Jun 2025   Prob (F-statistic):           4.06e-64
Time:                        16:48:13   Log-Likelihood:                -85.898
No. Observations:                 389   AIC:                             175.8
Df Residuals:                     387   BIC:                             183.7
Df Model:                           1                                         
Covariance Type:            nonrobust                                         
==============================================================================
                 coef    std err          t      P>|t|      [0.025      0.975]
------------------------------------------------------------------------------
const          2.0907      0.106     19.733      0.000       1.882       2.299
教练             0.5222      0.025     20.583      0.000       0.472       0.572
==============================================================================
Omnibus:                        7.237   Durbin-Watson:                   1.891
Prob(Omnibus):                  0.027   Jarque-Bera (JB):               11.232
Skew:                           0.036   Prob(JB):                      0.00364
Kurtosis:                       3.829   Cond. No.                         30.5
==============================================================================

Warnings:
[1] Standard Errors assume that the covariance matrix of the errors is correctly specified.
```

In [24]:

```
#说明教练的专业化水平对传播效果有显著的正向影响。
```

In [25]:

```
#中介效应检验 Mediating Effect of Consumption between Instructor Professionalism and Communication Outcomes
```

In [26]:

```
#总效应路径 a: 教练 → y
X_a = sm.add_constant(df1['教练'])
model_a = sm.OLS(y, X_a).fit()
print("路径 a（教练 → y 总效应）:")
print(model_a.summary())
```

```
路径 a（教练 → y 总效应）:
                            OLS Regression Results                            
==============================================================================
Dep. Variable:                      y   R-squared:                       0.523
Model:                            OLS   Adj. R-squared:                  0.521
Method:                 Least Squares   F-statistic:                     423.7
Date:                Wed, 04 Jun 2025   Prob (F-statistic):           4.06e-64
Time:                        16:49:01   Log-Likelihood:                -85.898
No. Observations:                 389   AIC:                             175.8
Df Residuals:                     387   BIC:                             183.7
Df Model:                           1                                         
Covariance Type:            nonrobust                                         
==============================================================================
                 coef    std err          t      P>|t|      [0.025      0.975]
------------------------------------------------------------------------------
const          2.0907      0.106     19.733      0.000       1.882       2.299
教练             0.5222      0.025     20.583      0.000       0.472       0.572
==============================================================================
Omnibus:                        7.237   Durbin-Watson:                   1.891
Prob(Omnibus):                  0.027   Jarque-Bera (JB):               11.232
Skew:                           0.036   Prob(JB):                      0.00364
Kurtosis:                       3.829   Cond. No.                         30.5
==============================================================================

Warnings:
[1] Standard Errors assume that the covariance matrix of the errors is correctly specified.
```

In [27]:

```
#说明教练的专业化水平对传播效果有显著的正向影响。
```

In [28]:

```
# 路径 b: 教练 → 中介_消费
X_b = sm.add_constant(df1['教练'])  # 自变量加常数
mediator = df1['中介_消费']          # 中介变量

model_b = sm.OLS(mediator, X_b).fit()
print("路径 b（教练 → 中介_消费）:")
print(model_b.summary())
```

```
路径 b（教练 → 中介_消费）:
                            OLS Regression Results                            
==============================================================================
Dep. Variable:                  中介_消费   R-squared:                       0.423
Model:                            OLS   Adj. R-squared:                  0.421
Method:                 Least Squares   F-statistic:                     283.6
Date:                Wed, 04 Jun 2025   Prob (F-statistic):           3.96e-48
Time:                        16:49:29   Log-Likelihood:                -271.30
No. Observations:                 389   AIC:                             546.6
Df Residuals:                     387   BIC:                             554.5
Df Model:                           1                                         
Covariance Type:            nonrobust                                         
==============================================================================
                 coef    std err          t      P>|t|      [0.025      0.975]
------------------------------------------------------------------------------
const          1.1738      0.171      6.879      0.000       0.838       1.509
教练             0.6881      0.041     16.840      0.000       0.608       0.768
==============================================================================
Omnibus:                       44.878   Durbin-Watson:                   1.871
Prob(Omnibus):                  0.000   Jarque-Bera (JB):               75.247
Skew:                          -0.710   Prob(JB):                     4.57e-17
Kurtosis:                       4.620   Cond. No.                         30.5
==============================================================================

Warnings:
[1] Standard Errors assume that the covariance matrix of the errors is correctly specified.
```

In [29]:

```
#说明教练的专业化水平对 “练习者的消费行为” 有显著的正向影响。
```

In [30]:

```
# 路径 c: 中介_消费 → y（控制教练）
X_c = sm.add_constant(df1[['教练', '中介_消费']])  # 自变量包含教练和中介变量
y = df1['y']  # 因变量

model_c = sm.OLS(y, X_c).fit()
print("路径 c（教练 + 中介_消费 → y）:")
print(model_c.summary())
```

```
路径 c（教练 + 中介_消费 → y）:
                            OLS Regression Results                            
==============================================================================
Dep. Variable:                      y   R-squared:                       0.548
Model:                            OLS   Adj. R-squared:                  0.546
Method:                 Least Squares   F-statistic:                     233.9
Date:                Wed, 04 Jun 2025   Prob (F-statistic):           2.86e-67
Time:                        16:49:58   Log-Likelihood:                -75.306
No. Observations:                 389   AIC:                             156.6
Df Residuals:                     386   BIC:                             168.5
Df Model:                           2                                         
Covariance Type:            nonrobust                                         
==============================================================================
                 coef    std err          t      P>|t|      [0.025      0.975]
------------------------------------------------------------------------------
const          1.9229      0.109     17.583      0.000       1.708       2.138
教练             0.4238      0.033     13.025      0.000       0.360       0.488
中介_消费          0.1429      0.031      4.648      0.000       0.082       0.203
==============================================================================
Omnibus:                        7.697   Durbin-Watson:                   1.886
Prob(Omnibus):                  0.021   Jarque-Bera (JB):               11.744
Skew:                           0.086   Prob(JB):                      0.00282
Kurtosis:                       3.834   Cond. No.                         43.8
==============================================================================

Warnings:
[1] Standard Errors assume that the covariance matrix of the errors is correctly specified.
```

In [31]:

```
#调节效应 Moderating Effect of Sports Type on Instructor Professionalism and Communication Outcomes
```

In [32]:

```
df1['教练*调节_类型'] = df1['教练'] * df1['调节_类型']
X_a_mod = sm.add_constant(df1[['教练', '调节_类型', '教练*调节_类型']])
mediator = df1['中介_消费']
model_a_mod = sm.OLS(mediator, X_a_mod).fit()
print("自变量->中介变量 路径 a 调节效应:")
print(model_a_mod.summary())
```

```
自变量->中介变量 路径 a 调节效应:
                            OLS Regression Results                            
==============================================================================
Dep. Variable:                  中介_消费   R-squared:                       0.447
Model:                            OLS   Adj. R-squared:                  0.443
Method:                 Least Squares   F-statistic:                     103.9
Date:                Wed, 04 Jun 2025   Prob (F-statistic):           2.85e-49
Time:                        17:13:38   Log-Likelihood:                -262.90
No. Observations:                 389   AIC:                             533.8
Df Residuals:                     385   BIC:                             549.7
Df Model:                           3                                         
Covariance Type:            nonrobust                                         
==============================================================================
                 coef    std err          t      P>|t|      [0.025      0.975]
------------------------------------------------------------------------------
const          1.2092      0.193      6.273      0.000       0.830       1.588
教练             0.6538      0.048     13.495      0.000       0.559       0.749
调节_类型          0.6483      0.434      1.493      0.136      -0.205       1.502
教练*调节_类型      -0.1045      0.102     -1.025      0.306      -0.305       0.096
==============================================================================
Omnibus:                       44.315   Durbin-Watson:                   1.917
Prob(Omnibus):                  0.000   Jarque-Bera (JB):               74.263
Skew:                          -0.703   Prob(JB):                     7.48e-17
Kurtosis:                       4.614   Cond. No.                         95.8
==============================================================================

Warnings:
[1] Standard Errors assume that the covariance matrix of the errors is correctly specified.
```

In [33]:

```
df1['教练*调节_类型'] = df1['教练'] * df1['调节_类型']
y = df1['y']
X_b_mod = sm.add_constant(df1[['教练', '调节_类型', '教练*调节_类型']])
model_b_mod = sm.OLS(y, X_b_mod).fit()
print("路径 b 调节效应:")
print(model_b_mod.summary())
```

```
路径 b 调节效应:
                            OLS Regression Results                            
==============================================================================
Dep. Variable:                      y   R-squared:                       0.532
Model:                            OLS   Adj. R-squared:                  0.529
Method:                 Least Squares   F-statistic:                     146.0
Date:                Wed, 04 Jun 2025   Prob (F-statistic):           3.56e-63
Time:                        17:14:00   Log-Likelihood:                -81.965
No. Observations:                 389   AIC:                             171.9
Df Residuals:                     385   BIC:                             187.8
Df Model:                           3                                         
Covariance Type:            nonrobust                                         
==============================================================================
                 coef    std err          t      P>|t|      [0.025      0.975]
------------------------------------------------------------------------------
const          1.9238      0.121     15.891      0.000       1.686       2.162
教练             0.5699      0.030     18.728      0.000       0.510       0.630
调节_类型          0.6376      0.273      2.338      0.020       0.101       1.174
教练*调节_类型      -0.1605      0.064     -2.506      0.013      -0.286      -0.035
==============================================================================
Omnibus:                        5.924   Durbin-Watson:                   1.902
Prob(Omnibus):                  0.052   Jarque-Bera (JB):                8.349
Skew:                          -0.049   Prob(JB):                       0.0154
Kurtosis:                       3.711   Cond. No.                         95.8
==============================================================================

Warnings:
[1] Standard Errors assume that the covariance matrix of the errors is correctly specified.
```

In [34]:

```
df1['中介_消费*调节_类型'] = df1['中介_消费'] * df1['调节_类型']
X_c_mod = sm.add_constant(df1[['教练',  '调节_类型', '教练*调节_类型','中介_消费','中介_消费*调节_类型']])
y = df1['y']
model_c_mod = sm.OLS(y, X_c_mod).fit()
print("X->中介变量->Y（调节作用在X->中介上，中介->Y）")
print(model_c_mod.summary())
```

```
X->中介变量->Y（调节作用在X->中介上，中介->Y）
                            OLS Regression Results                            
==============================================================================
Dep. Variable:                      y   R-squared:                       0.566
Model:                            OLS   Adj. R-squared:                  0.561
Method:                 Least Squares   F-statistic:                     100.0
Date:                Wed, 04 Jun 2025   Prob (F-statistic):           2.92e-67
Time:                        17:14:15   Log-Likelihood:                -67.252
No. Observations:                 389   AIC:                             146.5
Df Residuals:                     383   BIC:                             170.3
Df Model:                           5                                         
Covariance Type:            nonrobust                                         
===============================================================================
                  coef    std err          t      P>|t|      [0.025      0.975]
-------------------------------------------------------------------------------
const           1.8242      0.128     14.248      0.000       1.572       2.076
教练              0.5160      0.041     12.658      0.000       0.436       0.596
调节_类型           0.3149      0.281      1.122      0.263      -0.237       0.867
教练*调节_类型       -0.2315      0.072     -3.207      0.001      -0.373      -0.090
中介_消费           0.0824      0.043      1.905      0.058      -0.003       0.167
中介_消费*调节_类型     0.1450      0.062      2.345      0.020       0.023       0.266
==============================================================================
Omnibus:                        7.831   Durbin-Watson:                   1.895
Prob(Omnibus):                  0.020   Jarque-Bera (JB):               12.378
Skew:                           0.061   Prob(JB):                      0.00205
Kurtosis:                       3.865   Cond. No.                         140.
==============================================================================

Warnings:
[1] Standard Errors assume that the covariance matrix of the errors is correctly specified.
```

In [ ]:
