# Supplementary material for: Propagating wellness: understanding the communication mechanisms of yoga and Wuqinxi (five animal frolics) via Lasswell’s 5W framework
Source: Front Public Health. 2026 Jul 23;14:1850942. doi: 10.3389/fpubh.2026.1850942 (PMC13443314; doi:10.3389/fpubh.2026.1850942)
Supplement: Supplementary file 2 [file Data_sheet_1.zip › Supplementary_Materials/9Testing_for_Venue_Facilities.html]

场馆检验0606


In [1]:

```
import pandas as pd
import warnings
warnings.filterwarnings('ignore')
```

In [2]:

```
import os
import pandas as pd
dir_=r"C:\Users\admin\Desktop\1128第八次课"
os.chdir(dir_)
```

In [3]:

```
data1=pd.read_excel("五禽戏答卷(1).xlsx")
data2=pd.read_excel("瑜伽答卷(1).xlsx")
data1['type']=0
data2['type']=1
data2.columns = data1.columns
```

In [4]:

```
data = pd.concat([data1,data2])
```

In [5]:

```
data.columns
```

Out[5]:

```
Index(['序号', '所用时间', '来自IP', '1、您的性别是           。', '2、您的年龄是            。',
       '3、您的职业是            。', '4、您所在会馆的练习者人数为           。', '5、您练习该项目的时间有多长?',
       '6、您所在会馆的授课时间是         。', '7、您练习该项目是为了健康类目的，例如强身健体。',
       '8、您练习该项目是为了治疗类目的，例如产后恢复、治疗肩颈、脊柱侧弯等疾病。', '9、您练习该项目是出自对美与时尚的追求，例如减脂塑形。',
       '10、您练习该项目是为了促进社会交往，例如丰富业余生活。', '11、您练习该项目是为了缓解压力与改善心情。',
       '12、您认为观看该项目是美的享受。', '13、您认为练习该项目是美的体验。', '14、您认为您所在会馆提供了丰富多样的课程。',
       '15、您对您所在会馆提供的课程感到满意。', '16、您所在会馆的上课形式包括大班课（大于等于20人）。',
       '17、您所在会馆的上课形式包括小班课（10人以内）。', '18、您所在会馆的上课形式包括一对一私教课。',
       '19、您购买了与该项目相关的基本产品（如练功服）。', '20、您购买了与该项目相关的进阶产品（如练习器材等）。',
       '21、您购买了与该项目相关的指导产品（如书籍、视频资料等）。', '22、您没有购买与该项目相关的任何产品。',
       '23、您所在会馆的教练员接受过专业培训。', '24、您所在会馆的教练员拥有教练资格证书。',
       '25、您所在会馆的教练员拥有5年以上教学经验。', '26、您所在会馆的教练员是以教练为职业的专业工作者。',
       '27、您所在会馆的教练员定期进行培训、考核。', '28、您所在会馆的教学内容包括技术动作。',
       '29、您所在会馆的技术动作教学是多样的，根据不同的教学目标进行针对性的组合设计，不是固定套路的。',
       '30、您所在会馆的教学内容包括每一式的功理、功效、特点。', '31、您所在会馆的教学内容包括呼吸、意念。',
       '32、您所在会馆的教学内容包括除上述（技术动作，每一式的功理、功效与特点，呼吸，意念）外的其他内容。',
       '33、练习该项目，您感到自己的身体素质得到了显著提升（强身健体）。', '34、练习该项目丰富了您的业余生活。',
       '35、练习该项目帮助您扩大了社会交往圈。', '36、练习该项目满足了您对美与时尚的追求。',
       '37、练习该项目有效地缓解了您的压力，并改善了您的心情。', '38、您所在会馆的经费来源包括练习者交费。',
       '39、您所在会馆的经费来源包括企业资助。', '40、您所在会馆的经费来源包括政府资助。',
       '41、您所在会馆的经费来源除上述外，还包括其他来源。', '42、您对所在会馆的授课时间安排感到满意。',
       '43、您通过户外广告，了解到所在会馆的该项目。', '44、您通过互联网，了解到所在会馆的该项目。',
       '45、您通过书籍、报纸或杂志上的文章，了解到所在会馆的该项目。', '46、您通过观看相关比赛，了解到所在会馆的该项目。',
       '47、您通过朋友或熟人的口口相传，了解到了所在会馆的该项目。',
       '48、您所在会馆提供了丰富的基础设施配套（如更衣室、淋浴间、卫生间等）。',
       '49、您对所在会馆的基础设施（如更衣室、淋浴间、卫生间等）感到满意。', '50、您所在会馆重视会员对基础设施的反馈意见。',
       'type'],
      dtype='object')
```

In [6]:

```
import pandas as pd
import numpy as np
from factor_analyzer import FactorAnalyzer, calculate_bartlett_sphericity, calculate_kmo
import matplotlib.pyplot as plt
import seaborn as sns
```

In [7]:

```
columns = [ '15、您对您所在会馆提供的课程感到满意。','33、练习该项目，您感到自己的身体素质得到了显著提升（强身健体）。','34、练习该项目丰富了您的业余生活。', 
       '35、练习该项目帮助您扩大了社会交往圈。','36、练习该项目满足了您对美与时尚的追求。','37、练习该项目有效地缓解了您的压力，并改善了您的心情。'] 
df_dependent = data[columns]
```

In [8]:

```
#以上题项已通过因子检验，代表传播效果。
```

In [9]:

```
data['y']=df_dependent.mean(axis=1)
#对传播效果取平均值，赋值给data['y']
```

In [10]:

```
changguan = data[[ '48、您所在会馆提供了丰富的基础设施配套（如更衣室、淋浴间、卫生间等）。',
       '49、您对所在会馆的基础设施（如更衣室、淋浴间、卫生间等）感到满意。', '50、您所在会馆重视会员对基础设施的反馈意见。']]
```

In [12]:

```
# 初步因素分析
fa = FactorAnalyzer(rotation=None)
fa.fit(changguan)

ev, v = fa.get_eigenvalues()
# 查看方差解释率
variance = fa.get_factor_variance()

# 查看方差解释率
variance = fa.get_factor_variance()
# 获取实际因素数量
num_factors = min(len(ev), len(variance[1]), len(variance[2]))

# 构建表格
result_table = pd.DataFrame({
    '组件': [str(i + 1) for i in range(num_factors)],  # 动态生成组件编号
    '初始特征值_总计': ev[:num_factors],
    '初始特征值_方差百分比': [round(v * 100, 3) for v in variance[1][:num_factors]],
    '初始特征值_累积百分比': [round(v * 100, 3) for v in variance[2][:num_factors]],
    '提取载荷平方和_总计': ev[:num_factors],
    '提取载荷平方和_方差百分比': [round(v * 100, 3) for v in variance[1][:num_factors]],
    '提取载荷平方和_累积百分比': [round(v * 100, 3) for v in variance[2][:num_factors]],
})

result_table
```

Out[12]:

|  | 组件 | 初始特征值\_总计 | 初始特征值\_方差百分比 | 初始特征值\_累积百分比 | 提取载荷平方和\_总计 | 提取载荷平方和\_方差百分比 | 提取载荷平方和\_累积百分比 |
| --- | --- | --- | --- | --- | --- | --- | --- |
| 0 | 1 | 2.345325 | 68.826 | 68.826 | 2.345325 | 68.826 | 68.826 |
| 1 | 2 | 0.376655 | 1.710 | 70.536 | 0.376655 | 1.710 | 70.536 |
| 2 | 3 | 0.278020 | 1.076 | 71.612 | 0.278020 | 1.076 | 71.612 |

In [13]:

```
xiaofei = data[['19、您购买了与该项目相关的基本产品（如练功服）。', '20、您购买了与该项目相关的进阶产品（如练习器材等）。',
       '21、您购买了与该项目相关的指导产品（如书籍、视频资料等）。']]
```

In [14]:

```
#以上题项已通过因子检验，代表练习者的消费行为。
```

In [15]:

```
data['场馆']=changguan.mean(axis=1)
df1 = data[['场馆','y']]
df1['中介_消费']=xiaofei.mean(axis=1)
df1['调节_类型']=data['type']
```

In [16]:

```
## 假设检验
import pandas as pd
import statsmodels.api as sm
from statsmodels.stats.mediation import Mediation
from statsmodels.formula.api import ols
```

In [17]:

```
# 主效应检验 Overall Effect of Venue Facilities Completeness on Communication Effectiveness
X = sm.add_constant(df1['场馆'])  # 添加常数项
y = df1['y']  # 被解释变量

# 运行回归模型
model_h1 = sm.OLS(y, X).fit()
print(model_h1.summary())
```

```
                            OLS Regression Results                            
==============================================================================
Dep. Variable:                      y   R-squared:                       0.333
Model:                            OLS   Adj. R-squared:                  0.331
Method:                 Least Squares   F-statistic:                     193.3
Date:                Fri, 06 Jun 2025   Prob (F-statistic):           6.30e-36
Time:                        15:35:19   Log-Likelihood:                -150.92
No. Observations:                 389   AIC:                             305.8
Df Residuals:                     387   BIC:                             313.8
Df Model:                           1                                         
Covariance Type:            nonrobust                                         
==============================================================================
                 coef    std err          t      P>|t|      [0.025      0.975]
------------------------------------------------------------------------------
const          2.7195      0.111     24.400      0.000       2.500       2.939
场馆             0.3706      0.027     13.903      0.000       0.318       0.423
==============================================================================
Omnibus:                       14.877   Durbin-Watson:                   1.663
Prob(Omnibus):                  0.001   Jarque-Bera (JB):               33.936
Skew:                           0.046   Prob(JB):                     4.27e-08
Kurtosis:                       4.444   Cond. No.                         27.1
==============================================================================

Warnings:
[1] Standard Errors assume that the covariance matrix of the errors is correctly specified.
```

In [18]:

```
#说明场馆良好的基础设施对传播效果有显著的正向影响。
```

In [19]:

```
#中介效应检验 Mediating Effect of Consumption between Venue Facilities Completeness and Communication Outcomes
```

In [20]:

```
#总效应路径 a: 教学内容 → y
X_a = sm.add_constant(df1['场馆'])
model_a = sm.OLS(y, X_a).fit()
print("路径 a（场馆 → y 总效应）:")
print(model_a.summary())
```

```
路径 a（场馆 → y 总效应）:
                            OLS Regression Results                            
==============================================================================
Dep. Variable:                      y   R-squared:                       0.333
Model:                            OLS   Adj. R-squared:                  0.331
Method:                 Least Squares   F-statistic:                     193.3
Date:                Fri, 06 Jun 2025   Prob (F-statistic):           6.30e-36
Time:                        15:36:42   Log-Likelihood:                -150.92
No. Observations:                 389   AIC:                             305.8
Df Residuals:                     387   BIC:                             313.8
Df Model:                           1                                         
Covariance Type:            nonrobust                                         
==============================================================================
                 coef    std err          t      P>|t|      [0.025      0.975]
------------------------------------------------------------------------------
const          2.7195      0.111     24.400      0.000       2.500       2.939
场馆             0.3706      0.027     13.903      0.000       0.318       0.423
==============================================================================
Omnibus:                       14.877   Durbin-Watson:                   1.663
Prob(Omnibus):                  0.001   Jarque-Bera (JB):               33.936
Skew:                           0.046   Prob(JB):                     4.27e-08
Kurtosis:                       4.444   Cond. No.                         27.1
==============================================================================

Warnings:
[1] Standard Errors assume that the covariance matrix of the errors is correctly specified.
```

In [21]:

```
# 路径 b: 场馆设施 → 中介_消费
X_b = sm.add_constant(df1['场馆'])  # 自变量加常数
mediator = df1['中介_消费']          # 中介变量

model_b = sm.OLS(mediator, X_b).fit()
print("路径 b（场馆 → 中介_消费）:")
print(model_b.summary())
```

```
路径 b（场馆 → 中介_消费）:
                            OLS Regression Results                            
==============================================================================
Dep. Variable:                  中介_消费   R-squared:                       0.373
Model:                            OLS   Adj. R-squared:                  0.372
Method:                 Least Squares   F-statistic:                     230.4
Date:                Fri, 06 Jun 2025   Prob (F-statistic):           3.72e-41
Time:                        15:37:25   Log-Likelihood:                -287.38
No. Observations:                 389   AIC:                             578.8
Df Residuals:                     387   BIC:                             586.7
Df Model:                           1                                         
Covariance Type:            nonrobust                                         
==============================================================================
                 coef    std err          t      P>|t|      [0.025      0.975]
------------------------------------------------------------------------------
const          1.6466      0.158     10.402      0.000       1.335       1.958
场馆             0.5747      0.038     15.178      0.000       0.500       0.649
==============================================================================
Omnibus:                       39.405   Durbin-Watson:                   1.632
Prob(Omnibus):                  0.000   Jarque-Bera (JB):               64.638
Skew:                          -0.643   Prob(JB):                     9.21e-15
Kurtosis:                       4.527   Cond. No.                         27.1
==============================================================================

Warnings:
[1] Standard Errors assume that the covariance matrix of the errors is correctly specified.
```

In [22]:

```
#说明场馆良好的基础设施对 “练习者的消费行为” 有显著的正向影响。
```

In [23]:

```
# 路径 c: 中介_消费 → y（控制教练）
X_c = sm.add_constant(df1[['场馆', '中介_消费']])  # 自变量包含教练和中介变量
y = df1['y']  # 因变量

model_c = sm.OLS(y, X_c).fit()
print("路径 c（场馆 + 中介_消费 → y）:")
print(model_c.summary())
```

```
路径 c（场馆 + 中介_消费 → y）:
                            OLS Regression Results                            
==============================================================================
Dep. Variable:                      y   R-squared:                       0.424
Model:                            OLS   Adj. R-squared:                  0.421
Method:                 Least Squares   F-statistic:                     141.9
Date:                Fri, 06 Jun 2025   Prob (F-statistic):           6.25e-47
Time:                        15:38:15   Log-Likelihood:                -122.50
No. Observations:                 389   AIC:                             251.0
Df Residuals:                     386   BIC:                             262.9
Df Model:                           2                                         
Covariance Type:            nonrobust                                         
==============================================================================
                 coef    std err          t      P>|t|      [0.025      0.975]
------------------------------------------------------------------------------
const          2.2920      0.117     19.532      0.000       2.061       2.523
场馆             0.2215      0.031      7.066      0.000       0.160       0.283
中介_消费          0.2596      0.033      7.793      0.000       0.194       0.325
==============================================================================
Omnibus:                       16.671   Durbin-Watson:                   1.807
Prob(Omnibus):                  0.000   Jarque-Bera (JB):               35.334
Skew:                           0.177   Prob(JB):                     2.12e-08
Kurtosis:                       4.434   Cond. No.                         41.6
==============================================================================

Warnings:
[1] Standard Errors assume that the covariance matrix of the errors is correctly specified.
```

In [24]:

```
#调节效应  Moderating Effect of Sports Type on Adequacy of Venue Facilities and Communication Outcomes
```

In [25]:

```
df1['场馆*调节_类型'] = df1['场馆'] * df1['调节_类型']
X_a_mod = sm.add_constant(df1[['场馆', '调节_类型', '场馆*调节_类型']])
mediator = df1['中介_消费']
model_a_mod = sm.OLS(mediator, X_a_mod).fit()
print("自变量->中介变量 路径 a 调节效应:")
print(model_a_mod.summary())
```

```
自变量->中介变量 路径 a 调节效应:
                            OLS Regression Results                            
==============================================================================
Dep. Variable:                  中介_消费   R-squared:                       0.390
Model:                            OLS   Adj. R-squared:                  0.385
Method:                 Least Squares   F-statistic:                     81.94
Date:                Fri, 06 Jun 2025   Prob (F-statistic):           5.13e-41
Time:                        15:39:04   Log-Likelihood:                -282.18
No. Observations:                 389   AIC:                             572.4
Df Residuals:                     385   BIC:                             588.2
Df Model:                           3                                         
Covariance Type:            nonrobust                                         
==============================================================================
                 coef    std err          t      P>|t|      [0.025      0.975]
------------------------------------------------------------------------------
const          1.7857      0.182      9.806      0.000       1.428       2.144
场馆             0.5171      0.047     11.110      0.000       0.426       0.609
调节_类型          0.0952      0.419      0.227      0.820      -0.729       0.919
场馆*调节_类型       0.0199      0.098      0.204      0.838      -0.172       0.212
==============================================================================
Omnibus:                       36.577   Durbin-Watson:                   1.658
Prob(Omnibus):                  0.000   Jarque-Bera (JB):               64.242
Skew:                          -0.579   Prob(JB):                     1.12e-14
Kurtosis:                       4.619   Cond. No.                         88.4
==============================================================================

Warnings:
[1] Standard Errors assume that the covariance matrix of the errors is correctly specified.
```

In [26]:

```
df1['场馆*调节_类型'] = df1['场馆'] * df1['调节_类型']
y = df1['y']
X_b_mod = sm.add_constant(df1[['场馆', '调节_类型', '场馆*调节_类型']])
model_b_mod = sm.OLS(y, X_b_mod).fit()
print("路径 b 调节效应:")
print(model_b_mod.summary())
```

```
路径 b 调节效应:
                            OLS Regression Results                            
==============================================================================
Dep. Variable:                      y   R-squared:                       0.335
Model:                            OLS   Adj. R-squared:                  0.330
Method:                 Least Squares   F-statistic:                     64.73
Date:                Fri, 06 Jun 2025   Prob (F-statistic):           6.56e-34
Time:                        15:39:41   Log-Likelihood:                -150.28
No. Observations:                 389   AIC:                             308.6
Df Residuals:                     385   BIC:                             324.4
Df Model:                           3                                         
Covariance Type:            nonrobust                                         
==============================================================================
                 coef    std err          t      P>|t|      [0.025      0.975]
------------------------------------------------------------------------------
const          2.6480      0.130     20.411      0.000       2.393       2.903
场馆             0.3928      0.033     11.845      0.000       0.328       0.458
调节_类型          0.2244      0.299      0.752      0.453      -0.363       0.811
场馆*调节_类型      -0.0596      0.070     -0.857      0.392      -0.196       0.077
==============================================================================
Omnibus:                       14.845   Durbin-Watson:                   1.660
Prob(Omnibus):                  0.001   Jarque-Bera (JB):               34.129
Skew:                           0.022   Prob(JB):                     3.88e-08
Kurtosis:                       4.450   Cond. No.                         88.4
==============================================================================

Warnings:
[1] Standard Errors assume that the covariance matrix of the errors is correctly specified.
```

In [27]:

```
df1['中介_消费*调节_类型'] = df1['中介_消费'] * df1['调节_类型']
X_c_mod = sm.add_constant(df1[['场馆',  '调节_类型', '场馆*调节_类型','中介_消费','中介_消费*调节_类型']])
y = df1['y']
model_c_mod = sm.OLS(y, X_c_mod).fit()
print("X->中介变量->Y（调节作用在X->中介上，中介->Y）")
print(model_c_mod.summary())
```

```
X->中介变量->Y（调节作用在X->中介上，中介->Y）
                            OLS Regression Results                            
==============================================================================
Dep. Variable:                      y   R-squared:                       0.433
Model:                            OLS   Adj. R-squared:                  0.425
Method:                 Least Squares   F-statistic:                     58.41
Date:                Fri, 06 Jun 2025   Prob (F-statistic):           4.24e-45
Time:                        15:40:15   Log-Likelihood:                -119.48
No. Observations:                 389   AIC:                             251.0
Df Residuals:                     383   BIC:                             274.8
Df Model:                           5                                         
Covariance Type:            nonrobust                                         
===============================================================================
                  coef    std err          t      P>|t|      [0.025      0.975]
-------------------------------------------------------------------------------
const           2.1223      0.144     14.728      0.000       1.839       2.406
场馆              0.2405      0.038      6.266      0.000       0.165       0.316
调节_类型           0.2975      0.303      0.980      0.328      -0.299       0.894
场馆*调节_类型       -0.0366      0.074     -0.496      0.620      -0.182       0.108
中介_消费           0.2944      0.045      6.612      0.000       0.207       0.382
中介_消费*调节_类型    -0.0538      0.068     -0.791      0.429      -0.187       0.080
==============================================================================
Omnibus:                       16.204   Durbin-Watson:                   1.832
Prob(Omnibus):                  0.000   Jarque-Bera (JB):               37.675
Skew:                           0.097   Prob(JB):                     6.59e-09
Kurtosis:                       4.512   Cond. No.                         133.
==============================================================================

Warnings:
[1] Standard Errors assume that the covariance matrix of the errors is correctly specified.
```

In [ ]:
